# Towards an Aspect-Oriented Design and Modelling Framework for Synthetic Biology

**DOI:** 10.3390/pr6090167

**Published:** 2018-09-15

**Authors:** Philipp Boeing, Miriam Leon, Darren N. Nesbeth, Anthony Finkelstein, Chris P. Barnes

**Affiliations:** 1 Department of Computer Science, UCL, London WC1E 6BT, UK; 2 Department of Cell and Developmental Biology, UCL, London WC1E 6BT, UK; 3 Department of Biochemical Engineering, UCL, London WC1E 6BT, UK

**Keywords:** synthetic biology, CAD, mathematical modelling, host context, modularity, SynBioWeaver, aspect-oriented software engineering

## Abstract

Work on synthetic biology has largely used a component-based metaphor for system construction. While this paradigm has been successful for the construction of numerous systems, the incorporation of contextual design issues—either compositional, host or environmental—will be key to realising more complex applications. Here, we present a design framework that radically steps away from a purely parts-based paradigm by using aspect-oriented software engineering concepts. We believe that the notion of *concerns* is a powerful and biologically credible way of thinking about system synthesis. By adopting this approach, we can separate core concerns, which represent modular aims of the design, from cross-cutting concerns, which represent system-wide attributes. The explicit handling of cross-cutting concerns allows for contextual information to enter the design process in a modular way. As a proof-of-principle, we implemented the aspect-oriented approach in the Python tool, SynBioWeaver, which enables the combination, or weaving, of core and cross-cutting concerns. The power and flexibility of this framework is demonstrated through a number of examples covering the inclusion of part context, combining circuit designs in a context dependent manner, and the generation of rule, logic and reaction models from synthetic circuit designs.

## 1 Introduction

Synthetic biology aims to create a biological engineering field based on principles such as modularity and abstraction. Until now, it has predominantly used a component-based metaphor inspired by electronic circuits: a genetic part encapsulates a specific sequence of DNA associated with a particular function, and parts are the basic building blocks of genetic circuits [1,2]. This paradigm has enabled the construction of many systems over the past decade. However, for advanced clinical and industrial applications to be realised, we must first be able to design and build systems that can function predictably across a wide range of varying internal and environmental conditions. To some extent, many of the challenges that faced synthetic biology a decade ago have been addressed through better part design, the development of orthogonal part libraries and improved techniques for screening [3,4]. Despite these advances, the development of synthetic biology into a truly predictive engineering discipline remains a challenge.

The context of a heterologous system is the environment in which it resides. This can include the compositional context (sequence and parts), the host context and the environmental context (in which the host exists) [5]. A range of context dependent effects has been demonstrated in prokaryotic systems. Within a promoter, operator sequences and their relative positions can significantly affect transcription rates [6]. While ribosome sequence can be used to predict binding strengths [7], the relative position of both *cis* and *trans* sequences can alter transcription and translation [8,9]. Connecting modular parts to form combined systems faces further challenges including retroactivity, which alters system dynamics [10,11]. Host context alters protein synthesis efficiency evidenced by the need for codon optimisation of coding sequences [12] and heterologous system functionality is affected directly, through competition for resources such as ribosomes [13–17] and proteases [18], and indirectly, through the growth rate [19–21]. The environmental context, including growth media [22], temperature [23–25] and pH [26] are well known examples of modifiers of circuit behaviour. More complex effects are found under bioprocessing conditions [27,28] and the long-term evolutionary stability of plasmids depends on the homology of parts used [29–31], their propensity for mobile element insertion [32,33], and the burden on the host [17,34]. Context can also be exploited; two striking examples are creating growth dependent bistability [35] and synchronisation through post-translation coupling [36]. These examples demonstrate how contextual issues can interact across scales and intertwine with the modular parts that comprise the synthetic circuit. From a design point of view, one would like to handle context in a straightforward manner that interacts in a tractable way—be it to specify situations where particular genetic parts are needed, or dependencies between parts and environmental factors.

These examples demonstrate how contextual issues can interact across scales and intertwine with the modular parts that comprise the synthetic circuit. A simple example is the case of temperature; in addition to the Arrhenius dependence of reactions rates, temperature can indirectly alter a wide range of physiological processes such as RNA and protein synthesis, polysaccharide and fatty acid synthesis, flagella manufacture and solute uptake [37]. A full consideration of how temperature affects a synthetic circuit involves all the parts and modules comprising the circuit, and may require additional dynamical processes. The description of the circuit, which is the primary focus, becomes dependent on how temperature is included, destroying the modularity of the description. If we choose to change how temperature is represented, we must change the description of the whole system. This presents a challenge for any design framework that aims to capture context in a flexible manner.

**Figure 1:**
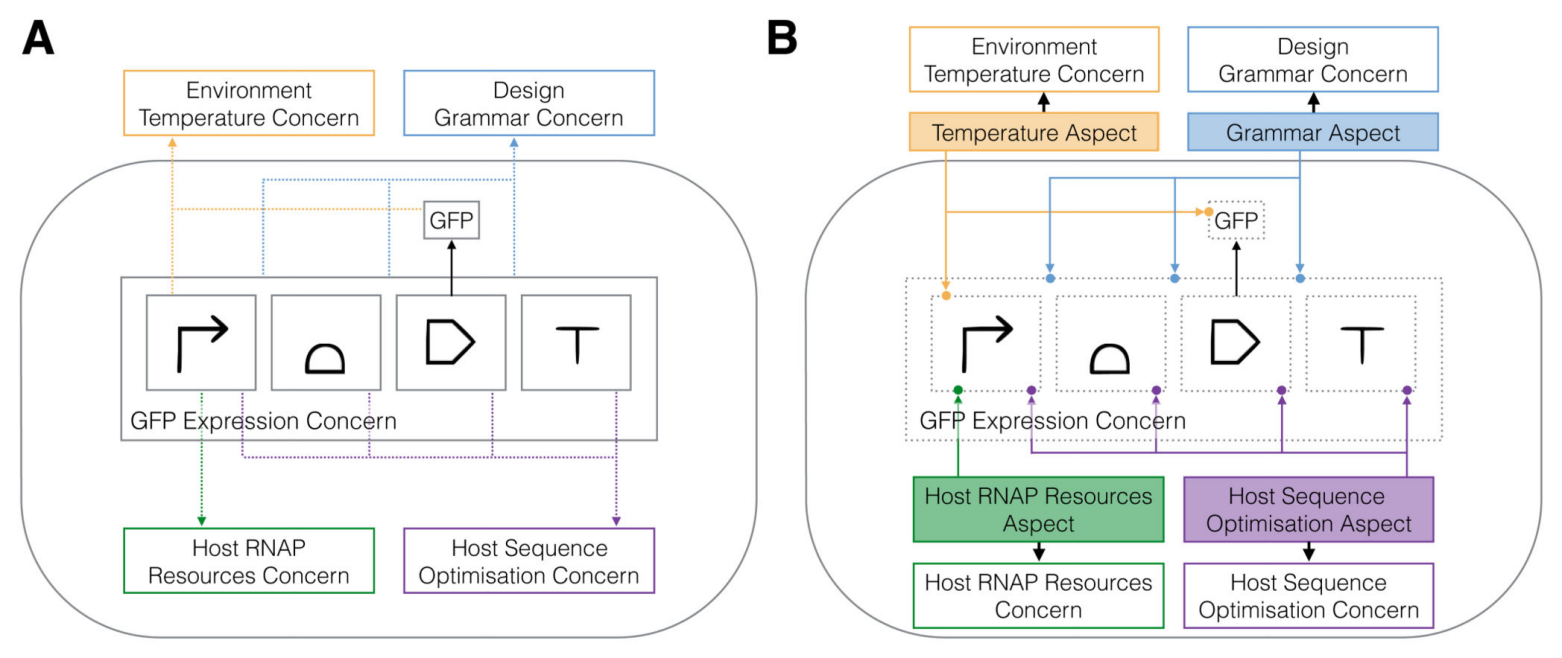
Example of core and cross-cutting concerns in synthetic biology. The core concern is the system expressing GFP (green fluorescent protein). (**A**) examples of cross-cutting concerns include contextual considerations such as the correct ordering of parts, environmental context such as temperature, and host context such as codon optimisation and cellular resources. These cross-cutting concerns are intertwined with the parts of the core concern; (**B**) aspect-oriented design allows for the modularisation of cross-cutting concerns.

Many developments in the field of software engineering are associated with the ability to partition or modularise elements of the system. One such form of partitioning is into “concerns”, defined as “specific requirements or considerations that must be addressed in order to satisfy an overall system goal” [38]. There are generally two categories of concerns: core concerns and cross-cutting concerns. Core concerns embody the main functionality of the system and cross-cutting concerns are often peripheral requirements that apply system-wide. In the temperature example, the core concern is the synthetic circuit and the cross-cutting concern is temperature. Whereas classical programming paradigms like procedural programming and OOP (Object Oriented Programming) are suitable paradigms to modularise core concerns, they struggle to modularise cross-cutting concerns. Instead, specifications dealing with cross-cutting concerns get tangled up in the implementation of core concerns (Figure 1A). Aspect Oriented Software Engineering (AOSE) was introduced to go beyond procedural programming and OOP to address the modularisation of all concerns, including cross-cutting concerns (Figure 1B).

Here, we introduce a design and modelling framework for synthetic biology that incorporates ideas from AOSE. Our design framework is unique in that cross-cutting concerns are explicitly handled and used to modularise contextual design issues. We outline the concepts underlying the framework and discuss how AOSE can help to modularise contextual concerns. We then introduce a proof-of-principle implementation of the framework in Python, called SynBioWeaver. The framework incorporates and builds upon many features of existing synthetic biology design tools. These include, but are not limited to, the constrained design of biologically valid devices in a manner similar to grammar based approaches [39,40] and hierarchical and modular modelling of biological systems [41–43]. The choice of implemention in Python enables seamless integration with existing software tools for rule-based modelling [44], Bayesian inference and design [45,46] and computing on GPUs [47]. Models can also be exported into SBML (Systems Biology Markup Language) [48] enabling integration with the many other tools supporting this standard. Finally, we demonstrate how simple contextual design concerns can be handled—and taken advantage of—in this framework by designing a switchable oscillator constructed from the coupling of a bistable switch and an oscillator via post-translational synchronisation.

## 2 Results

### 2.1 Concerns: A New Design Paradigm for Synthetic Biology

Concerns represent the conceptual parts of a system [49]. When these concerns are implemented and are clearly separated from each other, then that system is said to be *modular*. The core concerns represent the main aims of the system. For example, this may be the expression of a specific high-value protein, the sensing of an environmental signal, or oscillation in the concentration of a particular protein between two levels. Core concerns are set by the designer, are generally easily modularised and often hierarchical, meaning that they can be decomposed into smaller pieces which themselves can be considered core concerns. An example is the construction of a mult-input genetic logic gate [50]. One way to represent an arbitrary truth table is by combinations of NOR and NOT gates. These can be separated into different genetic logic gate subsystems, each implemented in a modular fashion, and could individually be core concerns depending on the design situation.

Our main contribution is to introduce the notion of cross-cutting concerns to synthetic biology. In contrast to core concerns, cross-cutting concerns are not easily modularised and can affect multiple parts of the system simultaneously. Contextual considerations generally fall into the category of cross-cutting concerns (Figure 1A). Examples include required part relationships for functional circuits, temperature and growth-rate dependence, competition for host resources, metabolic load and toxicity. We make no distinction between compositional (sequence and parts), host and environmental contexts and allow cross-cutting concerns to access all scales of the system. An important point is that cross-cutting concerns can be integral to the successful implementation of a system [36], which we will demonstrate through the post-translation coupling example (Section 2.8).

### 2.2 Aspect-Oriented Synthetic Biology

Aspect Oriented Software Engineering (AOSE) was introduced to address the modularisation of both core and cross-cutting concerns (Figure 1B). To understand how this is achieved in a software system, we must introduce the notion of *execution flow*. A computer program is traditionally executed by a single Algorithmic Logic Unit in a linear sequence of instructions that modify the state of memory stored in the computer’s registers. Execution flow is thus nothing more than a linear timeline, in which only a single thing happens at each point in time. Concurrency challenges this notion and adds multiple parallel timelines which require precise coordination. Aspect Oriented Programming (AOP) languages such as AspectJ, an extension of the Java language, achieve modularisation of cross-cutting concerns by introducing the following new constructs on top of established concepts such as classes and methods: *join points*, *point cuts* and *advice*. A *join point* is an “identifiable point in the execution of a program” [38], for example a method call or an object instantiation. A *point cut* is a construct that selects particular join points within the execution flow. Then, an *advice* can be used to define code that will be injected at particular point cuts. Point cuts and advice are bundled into class-like structures called *aspects*. An AOP compiler then uses a *weaver* that weaves in the advice code at the join points in the execution flow selected by the point cuts.

We believe that these ideas from AOSE are very useful when thinking about contextual design and abstraction in synthetic biology. There is not a unique mapping of execution flow to biological systems but we describe two levels at which these ideas can be applied. From the point of view of a genetic circuit design, within a coding sequence, there is a linear order of execution beginning with polymerase binding to the upstream promoter, followed by transcription and translation and ending in degradation. There are possible join points we can identify in this execution flow, such as the connections between parts at the transcriptional level. A point cut could select one of these, such as a point after a specific promoter, or it could select many of these join points, such as the set of points after all promoters. Mapping AOSE onto synthetic biology in this way can be used to modularise the concerns involved in the design and modelling of circuits. For example, we might define a point cut selecting those join points between a promoter and a protein coding region and weave in a ribosome binding site between them, thus fulfilling the design concern that “a ribosome binding site should lead to the binding of a ribosome that can then translate the protein coding region”. Further join points based on this level of scale include molecule join points (such as bonding, synthesis, degradation). Additionally, the framework can be expanded to include transcription and translation join points, for example in the following sequence: DNA part transcription to mRNA, mRNA translation to a polypeptide chain, chain folding to protein.

Another level at which AOSE ideas fit naturally is that of abstraction and model building. Take, for example, the area of whole cell modelling [51,52]. In case of the *M. genitalium* model, the simulation separates different cellular processes into 28 submodules. It assumes that in small time intervals (e.g., 1 s), modules can be run independently. Then, the modules update a list of cell variables to synchronise information. This process of independent modular simulation and subsequent information exchange is repeated until the cell divides (or a maximum number of simulation is reached). This is a model which defines a cyclic execution flow. In the simulation setup, an obvious join point is during the variable update step. In fact, the function of this join point can be described as weaving the different concerns of each module into the overall simulation, which satisfies all concerns. Each module independently—they can even be separate executing programs—implements a particular concern, under the assumption that cross-cutting is negligible for small time frames and that beyond this an exchange of information is sufficient. Additional join points could be considered at the point of variable transmission that would enable communication between modules without the need for the explicit weaving of code.

The identification of join points within a parts based genetic circuit is the approach that we adopt here. This is because genetic parts are the elements at the core of synthetic biology, used for construction and modification, and can thus be seen as “instructions” whose flow of execution can be manipulated. In addition, by placing join points at the parts and molecule level, we can represent contextual issues arising from part interactions with other parts (including, in principle, sequence level effects) and more global interactions due to host and environmental context. Although we do not fully implement the ideas of AOSE in model generation, we do allow for the generation of context dependent models.

### 2.3 The SynBioWeaver Framework

**Figure 2:**
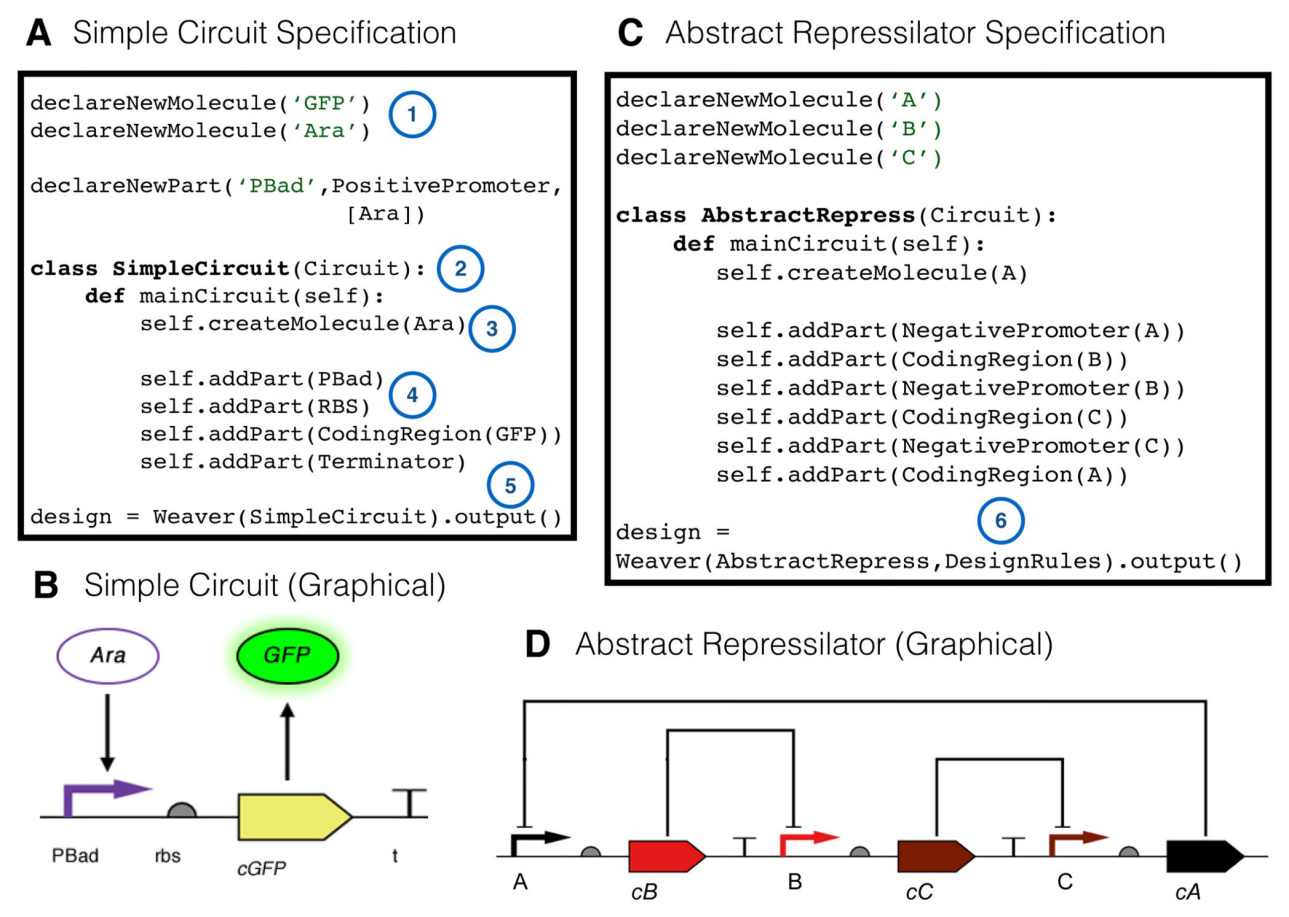
Specifiying basic circuits in the SynBioWeaver syntax. (**A**,**B**) code and graphical representation of a circuit coding for GFP induced by Ara; (**C**,**D**) code and graphical representation of an abstract repressilator circuit. 1: Types of molecules (e.g., transcription factors, proteins) can be created dynamically at the beginning of a SynBioWeaver specification. Parts can be given an inheritance structure. For example, the declared PBad promoter is of type PositivePromoter. PBad is also specified to be affected by one Molecule, the previously declared Ara (corresponding to Arabinose); 2: Each specification needs to declare at least one genetic circuit. These are specified as a class of type Circuit, which must define a mainCircuit method which the waever calls to begin compiling the circuit; 3: Because the PBad promoter reacts to the presence of the Ara molecule, it must be created in the scope of SimpleCircuit, so that the weaver can create the information link between Ara and PBad. This does not mean that Ara must necessarily be present in a simulation, but rather that the connection between PBad and a potential Ara molecule exists; 4: Parts are added using the Circuit’s addPart method; 5: The Weaver class is used to compile the specification. This creates an ordered list of parts and molecules, as well as components with a similar structure. Aspects can also add additional outputs to the weaver; 6: In this specification, the RBS (ribosome binding site) and terminator are filled in by the DesignRules aspect. An arbitrary number of aspects can be added to the weaver compilation after the initial circuit.

To demonstrate some of the features and advantages of thinking in terms of aspect-oriented design, we developed the tool SynBioWeaver in Python. We chose an existing language, rather than develop a novel domain specific language, because, although a custom language might be conceptually cleaner, and could offer a more streamlined interface for the user, Python is already a popular language in computational and systems biology. The way in which circuits are specified is simple, and we expect that individuals with no Python programming experience to be able to specify complex circuits (Figure 2). Additionally, by basing the framework in Python, all of Python’s existing featureset would be available to a proficient programmer, enabling development in a more established manner.

**Figure 3:**
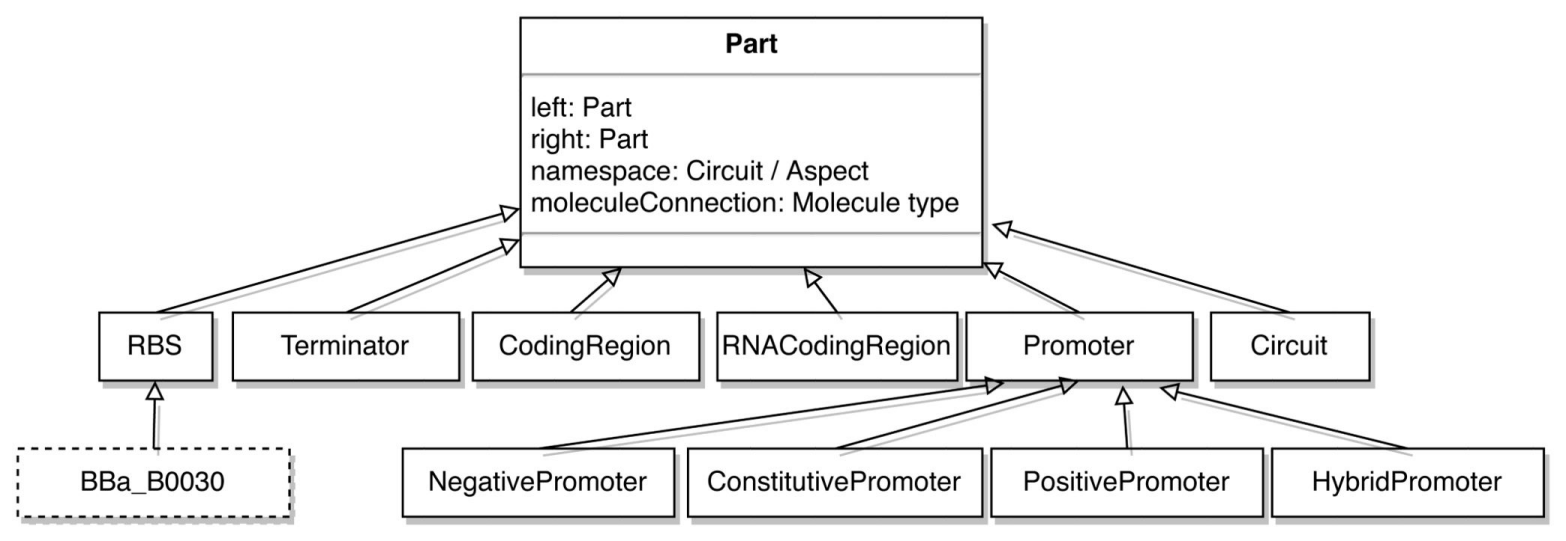
UML (Unified Modeling Language) class diagram shows the built-in part types in synbioweaver.core in solid boxes. Users can define new, more concrete part types, as shows by the BioBrick RBS part (dashed box).

To allow synthetic biology systems to be modelled in an aspect-oriented manner, SynBioWeaver provides classes to model genetic parts and molecules such as transcription factors. *Molecules* and *Parts* reside in *Circuits*, which can act as closed or semi-permeable compartments. A system can have a number of such compartments and sub-compartments and the level of encapsulation determined by the designer. Genetic parts as well as molecules follow a dynamic type hierarchy in the framework. Standard types, such as genetic part categories *Promoters*, *Ribosome binding site (RBS)*, *Coding Regions* and *Terminators*, with a shared parent *Part*, exist within a predefined hierarchy. New subtypes can be dynamically added by the user, such as a type referring to a specific BioBrick part (Figure 3).

**Figure 4:**
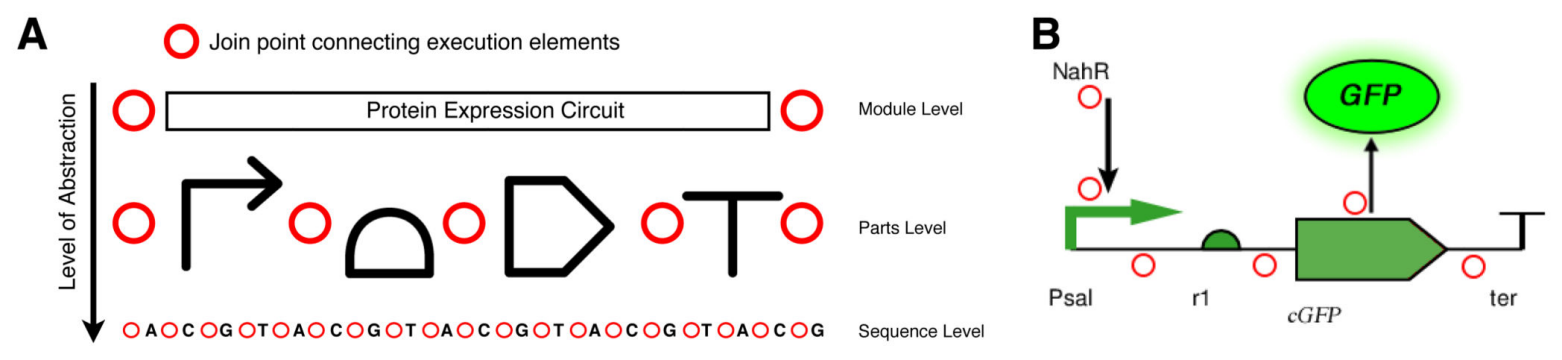
The possible join points in synthetic biology applications based on transcriptional circuits. (**A**) join points at the sequence, part and module level allows for hierarchal and modular design, abstraction and mathematical modelling; (**B**) the possible join points in a genetic circuit involving molecular interactions.

We loosely follow the AOP model of AspectJ and introduce synthetic biology relevant join points, point cuts and advice—bundled in aspects—to the framework. In our synthetic biology specific framework, we consider a genetic circuit made of parts as a sequence of execution steps, with join points between them (Figure 4A). A point cut is a construct that selects particular join points and an advice can be used to define code that will be injected at particular point cuts. Via this point cut advice system, the genetic circuit can be advised by influencing the execution flow. Point cuts and advice are bundled into class-like structures called aspects.

Another important ingredient of our framework is that parts can be enriched with *type advice*. These allow the inclusion of additional properties associated with the parts, which allows aspects to not only inject code at join points, but also to modify and add properties of parts in the system. Type advice is an extremely flexible way to build abstractions of the design such as mathematical models or computer code. Additionally, the mathematical models can be constructed in a context-dependent manner, which goes someway towards a full implementation of aspect-oriented model building.

**Figure 5:**
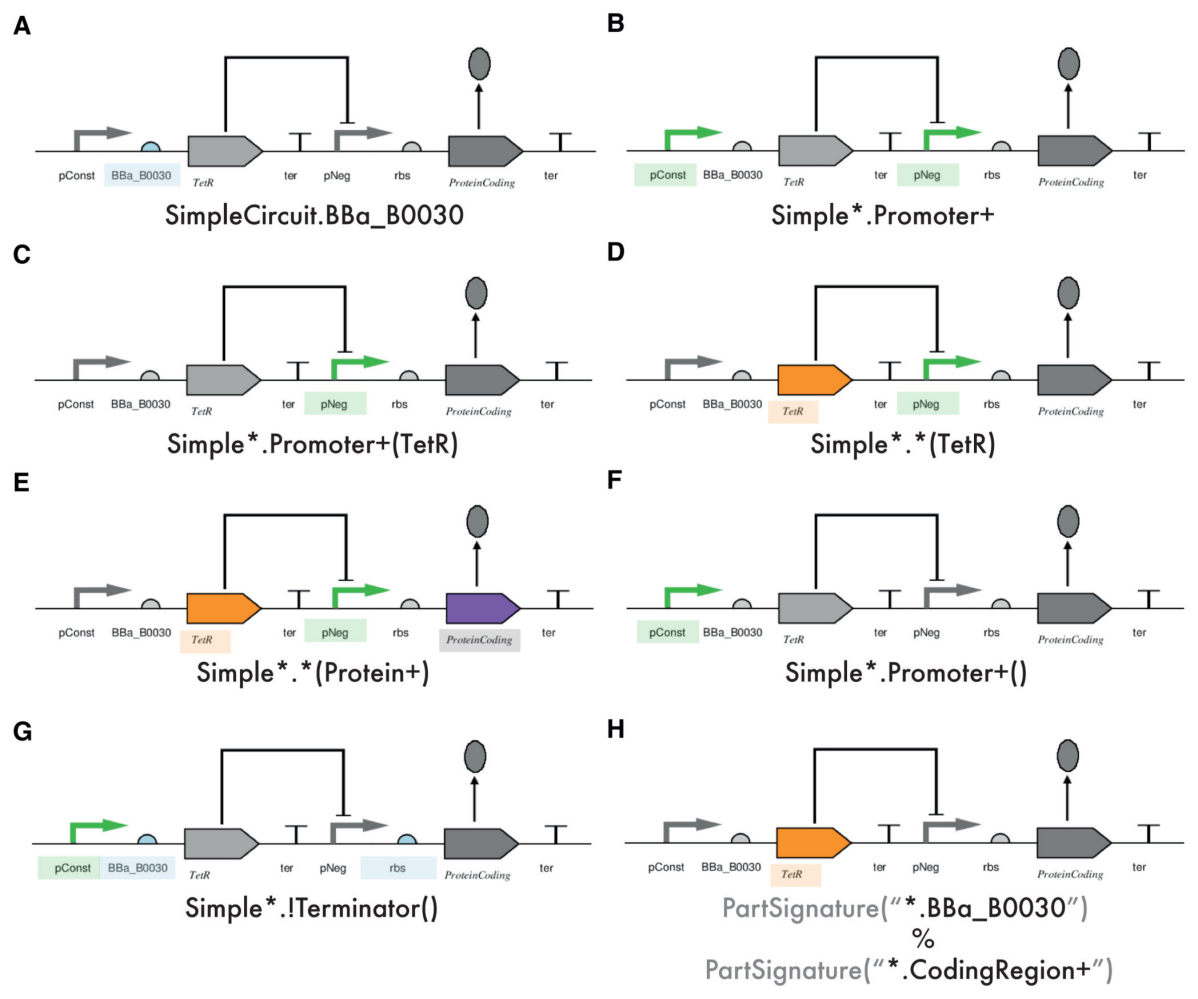
Overview of the possible part signatures. The syntax for the part signature is given at the bottom of each panel and the corresponding parts selected are shown in colour. (**A**) the part signature SimpleCircuit.BBa_B0030 selects the BioBrick part BBa_B0030 within SimpleCircuit; (**B**) here, the Promoter+ selects all promoters and subtypes. The Simple* part signature uses a wildcard (* symbol) to select any object of type Circuit whose typename begins with “Simple”; (**C**) the same as in (**B**) except that we now specify promoters that have Molecule associated of type TetR; (**D**) selects any object of type Part within any any object of type Circuit whose typename begins with “Simple”, and has a TetR Molecule associated; (**E**) selects any object of type Part which is associated with a Molecule of type Protein (and all derivatives); (**F**) selects any Promoter type, which is not associated with a Molecule (in this case the constitutive promoter); (**G**) here, the ! negates the part signature so this selects all parts that are **not** connected to a Terminator object. The final closed parentheses indicate that these parts should be unregulated; (**H**) this demonstrates a point cut expression. The % concatenates the two Part Signatures so that we select the part BBa_B0030 connected to a Part of type CodingRegion. The returned context is the TetR coding region.

The Python class Weaver carries out the insertion of the advice code at the join points in the execution flow selected by the point cuts. To achieve the weaving of code, a point cut matching mechanism for synthetic biology systems was created (Figure 5). For point cuts to be effective, they must precisely select a single join point, as well as be able to select a whole group of join points based on relevant features, such as part name, part type and part hierarchy. Here, the rich inheritance hierarchy of elements in the synthetic biology system becomes particularly relevant. A SynBioWeaver point cut has to be able to select the relevant join points in the synthetic biology system, such as the addition of parts to circuits, or the introduction of molecules. The point cut is specified by a formatted string, known as a *part signature*, that includes wildcard characters to, for example, allow matching all names starting with a particular sequence of letters or to select specific part types. This is accompanied by an operator to declare whether the advice code should be inserted before or after the element is introduced to the system, or whether the advice should replace the element. Furthermore, the point cut can be given a higher or lower priority, which can resolve conflicts if multiple point cuts match (Supplementary Figure S4). The *part signature* ends with the name of the part and an optional name of a molecule in parenthesis. Prior to the part name, the signature can be specified to only match parts that inhabit certain compartments. Compartments can be composed into multiple levels. In this case, compartment name matching works in a similar manner as accessing member variables within classes. Similarly, a *molecule signature* type exists that can be used to match molecules within the system. In the following, we demonstrate the viability of our approach, through a constructed a set of real world examples that exhibit a range of features and benefits of the framework.

### 2.4 A Simple Design Constraint Example

Figure 2 demonstrates how simple circuits can be specified in our framework together with the output in SBOL Visual, generated using an included additional output advice, which generates code for the Pigeon tool [53]. Here, we shall demonstrate more concretely the interaction between the main circuit design and an aspect that describes how design concerns can be expressed. The simple design constraint is that any coding region should be proceeded by an RBS and followed by a terminator sequence. Some existing tools, such as GenoCAD [39], approach this problem by defining a context free grammar which *checks* to see if the design is permitted. Here, we will demonstrate how SynBioWeaver takes a different approach by *modifying* the design so that it satisfies the concern.

**Figure 6:**
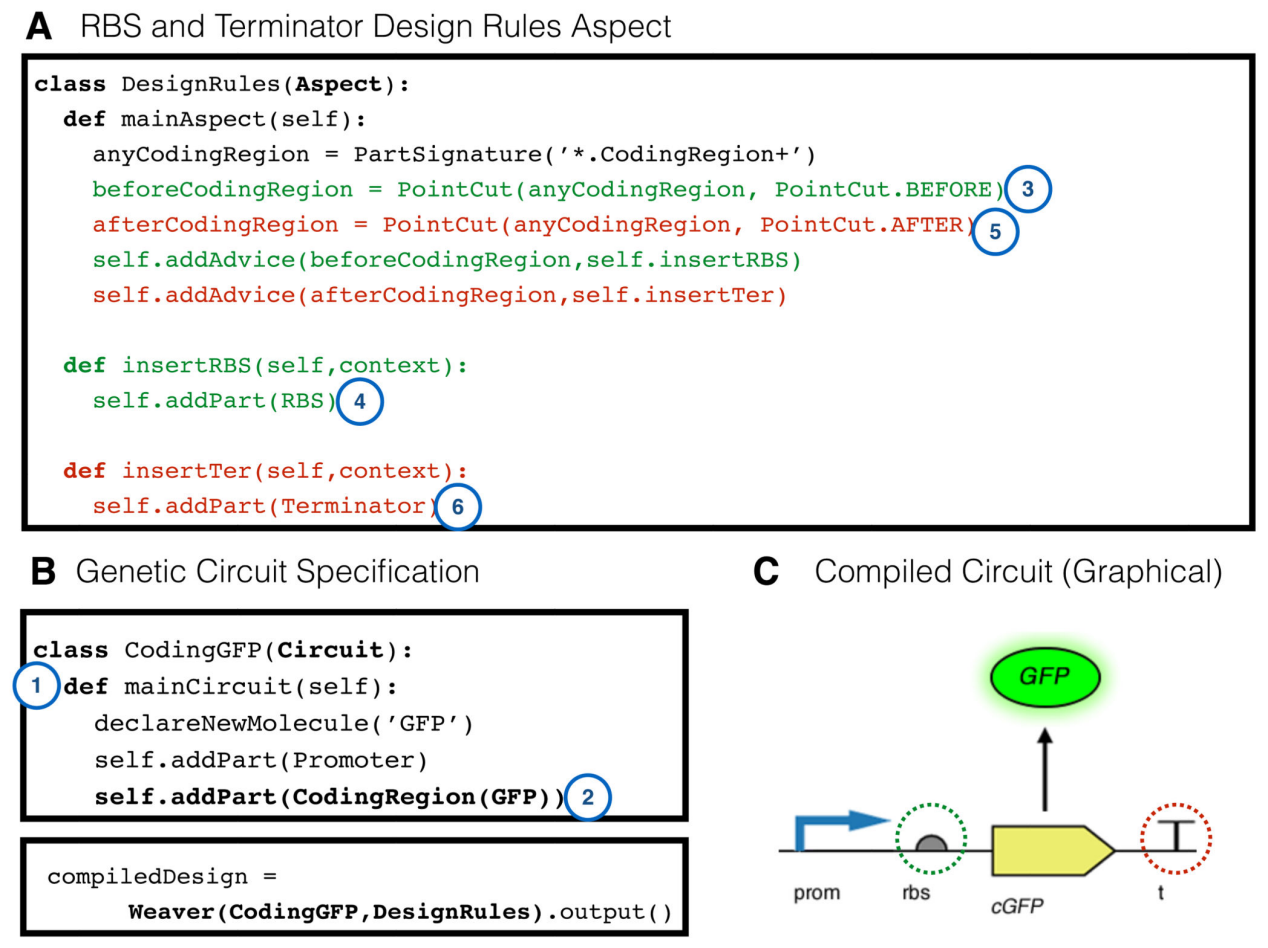
Design constraints example. (**A**) shows the specification of an aspect in SynBioWeaver, that encodes the concern that RBS and Terminator parts should be placed around a CodingRegion; (**B**) shows a specification for a GFP coding circuit, without RBS or Terminator parts, and the instruction to weave this CodingGFP circuit with the DesignRules aspect; (**C**) is a graphical representation of the compiled circuit. The Weaver first creates both the CodingGFP circuit and the DesignRules aspect. It begins weaving the circuit by calling the mainCircuit method (1). When the GFP coding region is added (2), the Weaver registers a matched point cut (3) and thus first executes insertRBS (4). Then, the coding region is added. Similarly, now the Weaver registers that second point cut is matched (5), so that insertTer is executed (6). In this simple example, generic RBS and Terminator parts are added to the circuit. However, the power of this approach is that specific contexts could be programmed—for example, selecting a terminator with minimal homology to others within the circuit.

Figure 6A illustrates an aspect called DesignRules. Selection of coding regions in the system is done with the PartSignature method. The * acts a wildcard so that all coding regions are selected (but this could be restricted, see the documentation). Then, two point cuts are defined, called beforeCodingRegion and afterCodingRegion which select the join point before and after any coding region, respectively. For the first point cut, advice is defined that inserts an RBS part whenever the point cut is encountered and similarly a terminator is inserted whenever the second point cut is encountered. Figure 6B shows how a simple gene circuit expressing GFP can be defined using the syntax of SynBioWeaver. An object called CodingGFP is created which inherits from the Circuit base class. A member function, mainCircuit, is defined which declares a new GFP molecule together with a coding region for the molecule plus a promoter. The weaver takes as an argument the DesignRules aspect and compiles it into the CodingGFP circuit. Figure 6C shows the output as SBOL Visual with RBS and terminator emphasised. This simple example is for demonstration only; it is not robust to the design already including an RBS or terminator, although a check could easily be programmed using type advice. However, it does demonstrate that using the genetic circuit join point model, design constraints can be enforced in a conceptually simple manner. Furthermore, genetic circuit design rules can be decomposed into reusable and easily customisable modular concerns.

### 2.5 Designs for Switchable Oscillating Systems Using Concerns at the Part and System Levels

**Figure 7:**
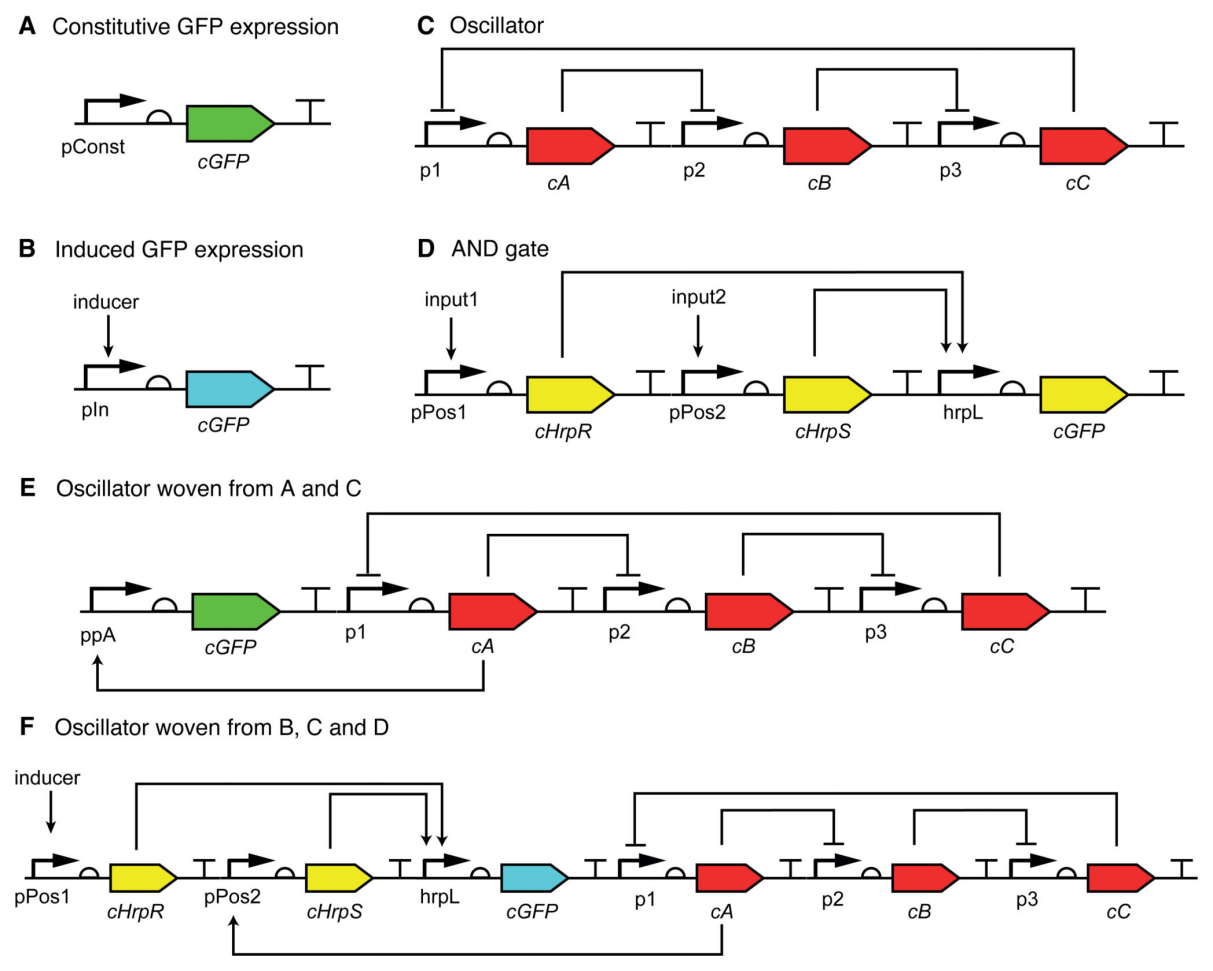
The switchable oscillator example. (**A**) a circuit constitutively expressing GFP; (**B**) expression of GFP is controlled via an inducer; (**C**) an oscillation circuit based on the repressilator; (**D**) an abstract AND gate which can link two inputs; (**E**,**F**) the result of weaving the circuits using an aspect that reacts to GFP coding sites and adds oscillation to its behaviour; (**E**) for the constitutively expressed circuit, the aspect only needs to replace the circuit’s promoter to link it to one of the transcription factors in the oscillation motif; (**F**) in the case of the induced GFP circuit, B, the aspect requires the AND gate to link the oscillation transcription factor to the original inducer.

The next example that we shall consider is a switchable oscillator system based around the repressilator [54]. This more complex example demonstrates a number of the advantages of thinking about system design in terms of concerns; the various levels that concerns can operate at (part and system level) and how system concerns allow for design reuse and combination. Figure 7 shows an outline of the example (the code is included in the package and explained in the Supplementary Information). In Figure 7A,B, there are two reporter concerns; a standard GFP reporter which is specified using a ConstitutivePromoter part, and, alternatively, a GFP reporter dependent on an external inducer (that is, an additional on-switch), using a PostivePromoter, pIn, induced by an inducer. Figure 7C shows an oscillation abstract gene regulatory network (using abstract, non-determined transcription factors). Figure 7C shows an AND gate concern based on that of Wang et al. [22].

The idea here is to generate a design for an oscillating GFP output by combining the oscillation circuit with a GFP reporter circuit. Therefore, an aspect is created, called Repressilation, that can weave the oscillation concern into either of the two output circuits. This can be done by making the promoter upstream of the GFP coding region dependent on one of the proteins from the oscillator. In the simple case that the promoter is unregulated, it must be replaced by one that is regulated by the oscillation output. However, if the promoter is already regulated, replacing it is not straightforward, since the dependence on inducer would be lost (we assume that there is no promoter than can be simultaneously induced by inducer and positively regulated by any protein within the oscillator). This is where the modular AND gate concern comes in. It can be combined with both the GFP output circuit and the oscillator circuits in order to preserve the original input. Therefore, the Repressilation aspect is able to change the promoter of the output circuit to make the output dependent on oscillation. Figure 7E,F show SBOL Visual representations of the final circuit in the case that the GFP output is constitutive and inducible, respectively. This example demonstrates how concerns at the part level can interact with concerns at the system level and the easy way with which previously defined modules can be reused and combined for more complex designs.

### 2.6 Rule-Based Modelling as an Aspect

**Figure 8:**
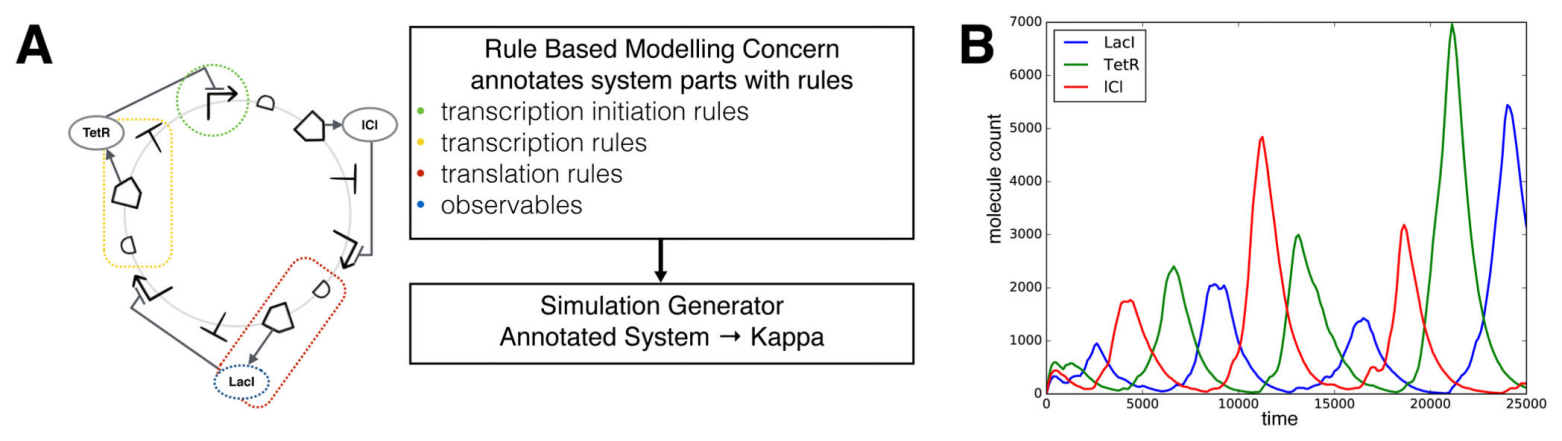
(**A**) an aspect implementing the rule based modelling concern annotates the Repressilator circuit with rules that can be used in a Kappa simulation. Different parts of the system (e.g., Promoters, Coding Regions) are found using specific point cuts, and rules are added using PySB. After annotation, a simulation file for Kappa can be generated; (**B**) example run of a stochastic kappa simulation file generated using SynBioWeaver’s rule based modelling aspect.

Rule-based modelling is an approach that has been gaining interest in both systems and synthetic biology as it eases the handling of complex systems governed by simple rules such as post-translational interactions and signalling networks. In rule-based modelling, a model is created as a collection of reaction rules in a rule-based modelling language such as Kappa [55,56]. Rules can be written to encapsulate chemical reactions, as well as transcriptional and translation processes, amongst others. The models can be used to derive systems of ODEs (ordinary differential equations) or can be simulated as a stochastic simulation. We created an aspect that can generate rule-based models by annotating the parts and molecules in the system with appropriate reaction-rules. Figure 8 gives an overview of how this aspect was used to create a stochastic rule based model of the repressilator [54].

The aspect defines rules for transcription factor binding to promoter sites, transcriptional and translational processes, and degradation processes. Within the aspect, the rules are specified in an abstract manner using PySB [44] and when the system is woven, the rules are instantiated for the given scenario. Using PySB’s export functionality, Kappa code for stochastic simulation is generated [55,56] (Figure 8A). Furthermore, PySB allows the export of the model to SBML for further integration with other modelling tools. The Kappa rules generated by our aspect contains only 19 abstract rules but were able to recreate the qualitative dynamics of the system (Figure 8B). The aspect is fully reusable and can be easily used to annotate other systems with specific rules for stochastic modelling. In addition, it could also be used to define a set of more complex abstract rules so that more detailed models can be generated.

### 2.7 Type Advice, Abstraction and Cross-Cutting Contextual Model Generation

We described above how AOSE approaches could in principle be be applied at a number of levels. The previous examples have focussed on the parts level and in this example we show how similar ideas can be applied to model generation and contextual considerations. The generation of models is done using type advice to access the different parts of the circuit. Initially a mapping between promoters, coding regions and regulatory interactions is constructed. This is then used to automatically generate the stoichiometry matrix, rate parameters and equations of the model. Clearly, the level of abstraction should be flexible to allow for different scenarios and we have provided three different aspects: mass action kinetics at the protein level, mass action kinetics including protein and RNA, and an abstraction based on the Shea–Ackers formalism including both RNA and protein reactions [57]. In principle, other user defined kinetic strategies could be implemented. For example, in the case of mass action kinetics, if a negative promoter is present, it will generate reactions consisting of its regulator reversibly binding to the promoter. For a coding region, it will search for the proceeding promoter and add a gene expression reaction for the promoter and the resulting coded protein. One parameter is defined for each reaction and the rates are calculated by assuming the law of mass action (by multiplying the parameter with the reactants of the corresponding reaction). Aspects are provided for printing out the resultant biochemical reactions and stoichiometry matrix, or for the generation of an SBML model [48,58].

**Figure 9:**
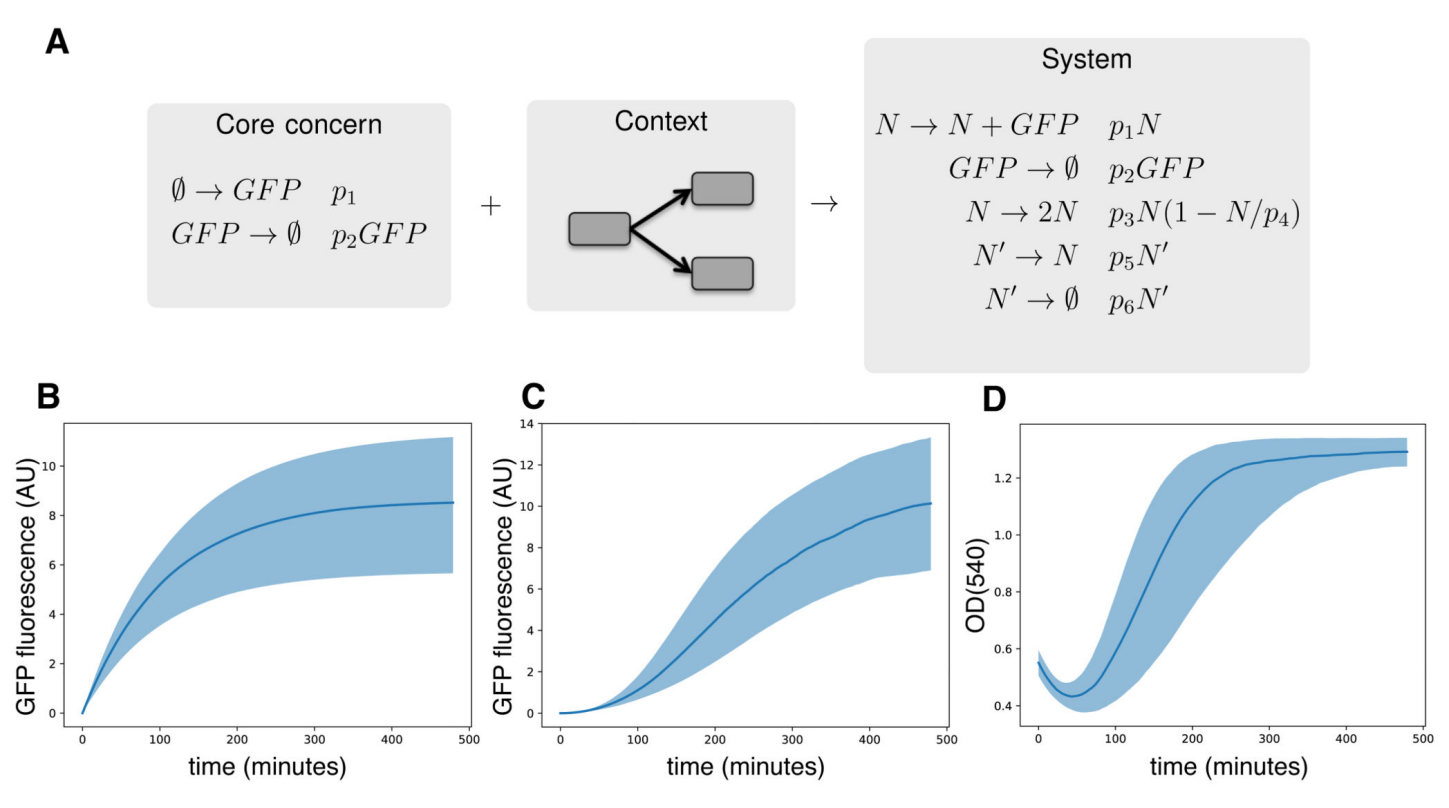
Models for the characterisation of parts. (**A**) the core concern is a promoter controlling expression of GFP, which can be placed in a cell growth cross-cutting concern [21,59]; (**B**) fluorescence time course simulations of the core concern only. The line represents the median time course and the shaded region the 0.9 probability region; (**C**,**D**) time-course simulations of fluorescence (**C**) optical density (**D**) of the complete system comprising core and cross-cutting concerns.

A unique feature of this approach is that contextual considerations can be modularised and here this is provided through contextual aspects that take the generated model and add additional contextual interactions. For example, when fitting growth and fluorescence curves, one must place the core concern(s) into the context of a reproducing bacterial population. (Figure 9). We provide three different contextual aspects: exponential growth, logistic growth and a model including a dying sub population of cells we term “lag logistic growth” [21,59]. An example of combining lag logistic growth with a simple model of constitutive GFP expression is given in Figure 9A. The transformed model can again be printed out or written to SBML, but we provide additional aspects that translate the above combined reaction system into CUDA and run simulations or perform Bayesian inference given some data. Figure 9B–D demonstrates the result of simulation of the core concern with and without the inclusion of the cross-cutting concern.

While there are other tools for characterisation and inference in synthetic biology [60], our approach logically separates the functionality of the circuit from the context that it resides in. This enables the synthetic biologist to place a single design into multiple contexts. These could range from laboratory conditions, different chassis, or industrially relevant scenarios [27]. The key point is that, despite these different contexts, the design remains invariant, it is only its abstraction that may change. To highlight this, additional examples within the package show how contextual interactions can be reversed engineered using Bayesian model selection [61], which could not only facilitate host and chassis optimisation but also discover new and interesting biological interactions. Of course, it may be advantageous to change the design based on particular contexts, changing a promoter depending on the pH or levels of CO_2_ for example, which could also be handled in our concern based framework.

### 2.8 Designing with Core and Cross-Cutting Concerns: Post-Translational Coupling of a Bistable Switch and an Oscillator

**Figure 10:**
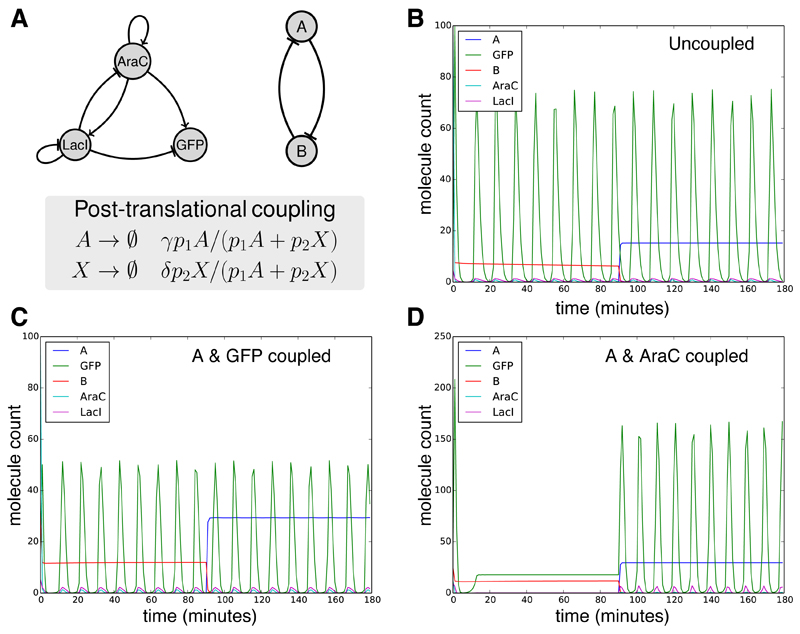
Post-translational coupling of a bistable switch and and oscillator (**A**)—the model setup. Both the oscillator and switch core concerns are based on previous designs [23,62,63], and defined as two sub compartments (separate Circuit objects) contained within the system. Additionally, a cross-cutting concern is defined that captures the post-translational coupling; one species in each sub circuit is tagged such that they share a common proteasome. In the case of the switch, the species is protein *A*, whereas, in the oscillator system, it can take one of two values *X* = {*GFP*, *AraC*}; (**B**) the uncoupled system with inducer added at *t* = 90 min. The system coupled through degradation of A and GFP (**C**) does not show robust bistable switching behaviour but coupling of A and AraC does (**D**).

A switchable oscillating system design was already presented, but this device would be susceptible to retroactivity [10,11], where the limit cycle of the oscillator is destroyed by the interaction with the AND gate. Another approach to achieving the same design goal has recently emerged, which takes advantage of queuing processes involved in the enzymatic degradation of proteins by proteases [18,36]. We used SynBioWeaver to examine whether the coupling of a bistable switch, consisting of two mutually inhibitory transcription factors [62,63], and a robust oscillator incorporating positive and negative feedback [23], could form a bistable oscillating system (Figure 10A).

The design was constructed by specifying two separate Circuit objects, one for each core concern. To explore post-translational coupling, a cross-cutting concern was defined that captured the essential properties of enzymatic degradation with one species in each sub circuit tagged such that they share a common protease (Figure 10A). The tagged protein from the switch was fixed to be protein A and we explored whether tagging GFP or AraC in the oscillator would be more effective in producing switchable oscillations (Figure 10C,D). Models were constructed using the Shea-Ackers modelling aspect. To parameterise the models, we used Bayesian inference to explore the parameter space giving rise to switching and oscillation behaviour [64,65] and obtained sets of parameters for each core concern. Figure 10B shows an example behaviour of the combined system with no direct coupling (growth rate dependent effects are neglected here but could in principle be included). We found that tagging both A and AraC could robustly produce a switchable oscillating system. Exploring the parameter sets capable of producing the desired behaviour, we found that stronger degradation of AraC by the protease was necessary (*δ >> γ* in Figure 10A). Initally, levels of A are low meaning that degradation of AraC is too high to cause oscillations. As the system switches to a high A state, A begins to bind to the protease and the amount of AraC in the system is increased, restoring the oscillator behaviour.

This example demonstrates how the overall system goal can be broken down into two core concerns (the oscillator and the switch) plus a cross-cutting concern (the coupling). Since the degradation term for a tagged protein depends on the concentrations of all tagged proteins in the system, by thinking in terms of core and cross-cutting concerns, this design can be represented in a modular and uncoupled fashion. We believe that this is the main advantage of thinking in terms of concerns. This example also shows that contextual issues can be integrated into the design process to take advantage of the context, rather than always engineering against it. By constructing a purely translational device, we would expose the design to the problem of retroactivity, which is avoided using this method. As we gain more understanding of fundamental biological processes, more ways to take advantage of context will emerge, and can be incorporated into the design framework.

## 3 Discussion

We have presented a novel framework based upon ideas from aspect-oriented software design and provided a proof-of-concept implementation in a Python tool, SynBioWeaver, which allows for a flexible and extensible tool kit for the design and modelling of biological systems. The advantages of this framework were demonstrated through part context examples, combining circuit designs in a context dependent manner, and the generation of rule, logic and reaction models from synthetic circuits. We also showed how bacterial growth dynamics can be represented by a cross-cutting concern and how this can be used to simulate real growth dynamics while leaving the original design (core concern) invariant. To demonstrate how cross-cutting concerns can be essential to system synthesis, we used SynBioWeaver to design a switchable oscillating system composed of a post-translationally coupled switch and oscillator.

Contextual design considerations are handled by a number of existing tools either directly or indirectly. For example, the GenoCAD language, which is based on a context-free grammar with parts as terminals, aims are to reduce design errors by specifying a grammar that formalises rules for the composition of parts [39]. Because it enforces combinations of parts that will generate a biologically valid construct, the grammar encodes the contextual requirements of the parts. Both GEC [40] and Eugene [66] are languages that allow the specification of genetic circuits involving abstract parts which can then be compiled into potentially multiple realisations satisfying some design constraints. These tools focus on the core concern of the genetic circuit design though in some sense the design constraints, in the form of regulatory interactions between abstract parts, quantify the context of the *required* parts. Proto is an open source functional programming language for spatially distributed computation [67] and is an integral part of the TASBE workflow that encompasses the complete synthetic biology design process [68]. TASBE does have the ability to accept contextual information on the cellular platform, which is read in before compilation and contains information on the available design motifs for the particular cellular context. In this work, we emphasised that the separation of core concerns from cross-cutting concerns allows for the modularisation and incorporation of contextual design issues in a way that current frameworks cannot. We believe that considering system synthesis in this manner will expedite the design workflow and allow for more faithful design processes.

The main disadvantages to an aspect-oriented approach are two-fold. Firstly, a synthetic biological device is fundamentally a physical system governed by stochastic processes. Many processes occur between two points in time, and these are not precisely predictable. Therefore, the notion of an execution flow is much less straightforward in comparison to computing. Indeed, this raises the fascinating question of how biological systems perform computation in a parallel and robust manner, an active area of research [69]. Secondly, because of the nature of how the weaving and advice interact, it can be difficult to predict the computational algorithmic complexity of particular combinations of advice specifications. How consistency across different aspects is maintained, such as those that modify the design itself or a derived abstraction, is an interesting area for further work, and not just limited to aspect-oriented approaches to synthetic biology. Future work in this area could look at how different mappings of execution flow could impact the interaction of concerns. While the whole cell model approach seems too low-level to currently form the basis of a useful aspect-oriented synthetic biology framework, the idea seems worthy of exploration and could lead to approaches that allow the reuse of model components and thus cut development time.

In order to maximise the applicability of SynBioWeaver, many extensions should eventually be added. We limited ourselves to the part level and did not provide any aspects related to sequence context, despite this being an important area [4]. Circuit designs are currently output only in textual format and we plan to integrate directly with programmatic tools such as DNAPlotlib [70]. Although the framework implements abstract gene regulatory networks and instantiation of them, the approach is simple and cannot currently explore combinatorial design on a large scale in an efficient manner [66,68], which would require further study of the complexity. On a more practical level, to use the framework, users have to write short Python programs which can be a deterrent, although import of models from SBOL [71] combined with inbuilt aspects could reduce the need for Python programming.

In the future, we believe that by building on existing automated design approaches [64,68,72–75], such a framework can implement design generation and augmentation that can choose sequence and parts based on homology, known contextual relations, and quantitative part characterisation data [9]. This could drastically reduce combinatorial design space by producing contextually sound designs. Altering circuit designs based on host chassis and environmental conditions has obvious implications for bioprocessing and therapeutic applications [76]. To enable this approach to system design requires accurate knowledge of part parameters which is itself a challenge and will require a combination of standardisation [2,77–79], and new approaches that leverage sequence data [80], microfludic platforms [81], optimal experimental design [82] and Bayesian statistics [46,83].

Part-based thinking in synthetic biology is going to be central to engineering future biological systems. However, we believe that it is precisely the importance of cross-cutting concerns—here, the context in which the synthetic circuit resides—that justifies a formal approach to their modularisation. We have provided an important step towards this goal, though this is clearly an exciting and open field.

## 4 Materials and Methods

- SynBioWeaver is implemented in the Python package synbioweaver, licensed under the MIT licence and available on GitHub: https://github.com/ucl-cssb/synbioweaver
- The documentation is accessible at: http://synbioweaver.readthedocs.org
- The functionality described here is implemented within the examples of the package. Rule-based modelling requires that PySB and KaSim are installed. Biochemical network simulation on GPUs requires CUDA, PyCUDA [84] and cuda-sim. Bayesian inference requires ABC-SysBio.

## Supplementary Material

**Supplementary Materials:** The following are available online at http://www.mdpi.com/2227-9717/6/9/167/s1.

Supplementary Information
