# Supplementary material for: Towards an Aspect-Oriented Design and Modelling Framework for Synthetic Biology
Source: Processes (Basel). Author manuscript; Available in PMC 2018 Dec 17. (PMC6296438; doi:10.3390/pr6090167)
Supplement: Supplementary Information [file NIHMS80853-supplement-Supplementary_Information.pdf]

Towards an aspect-oriented design and modelling framework for synthetic biology - Supplementary Information

Philipp Boeing, Miriam Leon, Darren Nesbeth, Anthony Finkelstein
and Chris P. Barnes

Contents

1 SynBioWeaver Implementation **2**

1.1 Overview 2
1.2 Simple Example: RBS and Terminator Design Rules 2
1.3 Circuitry Architecture 3
1.3.1 Parts 3
1.3.2 Molecules 3
1.3.3 Dynamically creating Part and Molecule types 3
1.3.4 Circuits 3
1.3.5 Circuit as Composites 4
1.4 Point Cuts 4
1.4.1 Part Signature 4
1.4.2 Point Cut Expressions 5
1.5 Aspects and Advice 5
1.5.1 Advice precedence 6
1.6 Type Advice 6
1.7 Weaving 7

2 A switchable oscillating system based on the repressilator combined with an AND gate **8**

2.1 Defining the concerns 8

3 Rule-based modelling as an output concern **11**

3.1 Overview 11
3.2 Defining the Concerns 11

4 Generating reaction network models and adding context **15**

5 Bayesian modelling and characterisation **16**

5.1 Code for constitutive GFP and different bacterial growth contexts 16
5.2 Model for the core concern plus lag logistic growth 17

6 Post-translational coupling of a bistable switch and an oscillator **17**

6.1 Code for the combined and coupled systems 17
6.2 Toggle switch model 19
6.3 Stricker oscillator model 20

1 SynBioWeaver Implementation

1.1 Overview

SynBioWeaver is a framework written in Python 2.7 that allows a synthetic biology system to be built in an aspect-oriented manner. To achieve this, SynBioWeaver provides classes to model genetic parts. Via a point cut system, the genetic circuit can be advised by influencing the execution flow. Furthermore, parts can be enriched with type advice to include additional properties, such as modelling rules. SynBioWeaver is able to combine all these advice into a finished design, which includes an ordered list of parts representing the genetic circuit. All classes are in the `synbioweaver.core` Python module.

All the code and examples are downloadable as the module `synbioweaver` from GitHub and have online documentation at the following urls:

- `https://github.com/ucl-cssb/synbioweaver`
- `http://synbioweaver.readthedocs.org`

1.2 Simple Example: RBS and Terminator Design Rules

To give an initial flavour for the framework, the following code listing shows a simple example. It defines a standard genetic circuit: a promoter, followed by an RBS, followed by a coding region for GFP, followed by a terminator. However, in this example the core-concern of expressing GFP under a Promoter has been separated from the cross-cutting concern of always surrounding a coding region with an RBS and a terminator.

Listing 1: SynBioWeaver Example: Design Rules

```python
from synbioweaver import *

declareNewMolecule('GFP')

class CodingGFP(Circuit):
    def mainCircuit(self):
        self.addPart(Promoter)
        self.addPart(CodingRegion(GFP))

class DesignRules(Aspect):
    def mainAspect(self):
        anyCodingRegion = PartSignature('*.CodingRegion+')

        beforeCodingRegion = PointCut(anyCodingRegion,PointCut.BEFORE)
        afterCodingRegion = PointCut(anyCodingRegion,PointCut.AFTER)

        self.addAdvice(beforeCodingRegion,self.insertRBS)
        self.addAdvice(afterCodingRegion,self.insertTerminator)

    def insertRBS(self,context):
        self.addPart(RBS)

    def insertTerminator(self,context):
        self.addPart(Terminator)

compiledDesign = Weaver(CodingGFP,DesignRules).output()
print compiledDesign
```

In the following sections, the classes used in the example will be described in detail. This includes *Part* and *Circuit* types, *point cuts* and *advice*, and the *weaver*. These relate to the two most high priority requirements:

- Circuits can be modelled as a list of parts. Parts are objects with meaningful types
- A weaver allows circuit design to be advised by design aspects

Some additional concepts are not described further, but can be found in the documentation, which also includes tutorials and examples.

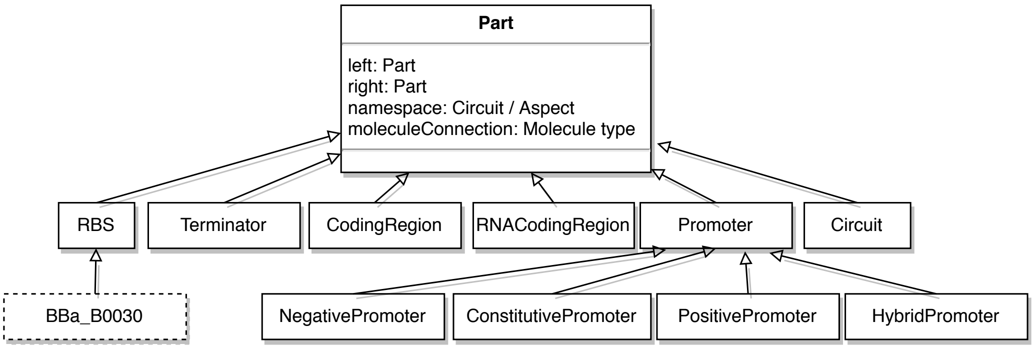

Figure S1: UML class diagram shows the built in part types in `synbioweaver.core` in solid boxes. Users can define new, more concrete part types, as shows by the BioBrick RBS part (dashed box).

1.3 Circuitry Architecture

1.3.1 Parts

Parts follow a rich inheritance hierarchy, with abstract parts of types such as "RBS" at the top, which could be subclassed into a concrete part such as "BBa_B0030"[1]. Figure S1 shows all part types in `synbioweaver.core`. Parts could have been made to differ via object attributes rather than type, however, since Python allows new types to be created quickly and dynamically we believe that it is conceptually clearer to represent each distinct part type (in biology) by a distinct type in the framework.

1.3.2 Molecules

Some parts, such as regulated promoters or coding regions are connected to molecules. These can be proteins such as GFP, transcription factors or other chemical agents interacting with the system. Unlike parts, molecules are always only used as types in SynBioWeaver, never objects. This is because they represent a class of Molecule rather than a concrete, physical molecule. In other words, if a promoter is regulated by a certain protein, it is regulated by all proteins of the type, not just by a single instance. All molecules are of subtype `Molecule`.

1.3.3 Dynamically creating Part and Molecule types

Two convenience functions, `declareNewPart()` and `declareNewMolecule()` are provided by SynBioWeaver. As seen in the example, they export the string name of the new type to the namespace. More information can be found in the documentation.

Listing 2: SynBioWeaver example of new Part and Molecule type declaration

```
# Protein is a subtype of Molecule
declareNewMolecule('GFP',Protein)

# GFP is now a new type in this namespace
declareNewPart('GFPcoding',CodingSite,GFP)

# a new part type has been created, getCodingFor returns a list of coding molecules
assert(GFPcoding().getCodingFor()[0] == GFP)
```

1.3.4 Circuits

A circuit is an ordered collection of parts. Circuits are given an `addPart()` method, and a mandatory `mainCircuit()` method, at which the circuit's execution begins. This design decision makes the circuit more

[1]A BioBrick part from the Registry of Standard Biological Parts, in the RBS category. `http://parts.igem.org/Part:BBa_B0030`

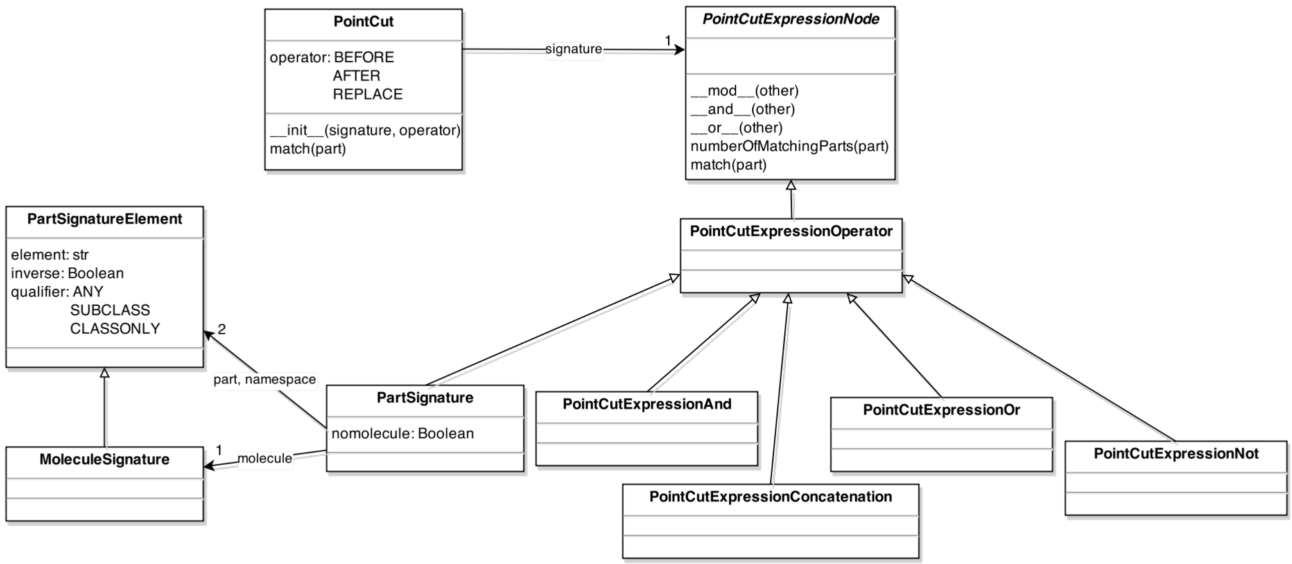

Figure S2: UML class diagram of classes involved in the Point Cut mechanism

like a dynamic programmatic construct, rather than a specified list. Indeed, all normal Python code can be used, such as loops and method calls to construct the genetic circuit with `addPart()` calls. The `Circuit` class is instantiated by the weaver, which then calls its `mainCircuit()` method. Whenever the circuit calls `addPart()`, the method is passed on back to the weaver, which decides if any point cuts are relevant. Thus each call to `addPart()` is an SynBioWeaver join point.

1.3.5 Circuit as Composites

As seen in the `Part` inheritance diagram (Figure S1), circuits are themselves parts. This means they can be used as composites, which reflects the idea of composite parts from the Registry of Standard Biological Parts. If a circuit is used as a composite, its `mainCircuit()` method is called when it is added via `addPart()`.

1.4 Point Cuts

A point cut in SynBioWeaver has to be able to select join points in the genetic circuit execution flow, i.e. calls to `addPart()`. Inspired by AspectJ, point cuts should be able to precisely select a single join point, or be able to select whole groups of join points based on features such as type name or type hierarchy. A SynBioWeaver `PointCut` object consists of an expression to select certain parts - the parameter to the `addPart()` call, and an operator to declare whether the advice should be inserted before or after the part is added, or whether the advice should replace the part. All classes involved in the point cut mechanism are shown in Figure S2.

1.4.1 Part Signature

A part signature is a single string that can match a part or group of parts. It consists of three elements. Firstly, it can specify a *namespace* of a `Part`, i.e. the circuit or aspect which has added it. Secondly, it can specify the part type. Thirdly, it can optionally specify a molecule the part should be connected to. Elements are matched by typename. A `+` at the end of each element specifies that all subclasses should be matched, `*` at the end of an element acts as a string wildcard, i.e. all types whose names begin with the characters preceding the wildcard will be matched. Each element can be inverted using a `!` at the beginning. 1 shows examples of valid and invalid signatures. Strings are validated and split using regular expressions, and the overall `PartSignature` object is implemented using classes for each element, so that matching a signature becomes the conjunction of matching the three elements.

Part Signatures	Matched Parts
SimpleCircuit.BBa_B0030	Parts added in SimpleCircuit of type BBa_B0030.
Simple*.NegativePromoter+(TeTR)	Parts of type NegativePromoter (including sub types) added in Aspects or Circuits whose typename begins with “Simple”, which have a moleculeConnection of type TeTR
!SimpleAspect.*(Protein+)	Any Part with a connection to a Protein added by an Aspect or Circuit whose name is not “SimpleAspect”
.(!TetR)	Any Part from any Circuit or Aspect whose moleculeConnection is not TeTR
*.Promoter+()	Any Part of (sub)type Promoter, whose moleculeConnection is None.

Table 1: Examples of Part Signatures

1.4.2 Point Cut Expressions

Often, a single part signature will be sufficient to define a point cut, but sometimes more control is needed. SynBioWeaver allows part signatures to be building blocks of more complex expressions, which represent an Abstract Syntax Tree (AST) of part signatures. Part signatures can be combined using boolean-or and boolean-and. A signature can also be concatenated to another signature, so that we can define join points based on a number of preceding parts. Lastly, a signature can be inverted.

`PointCutExpressionOr`, `PointCutExpressionAnd` and `PointCutExpressionConcatenate` nodes can be created using overloaded operators `|`, `&` and `%`, respectively. Inversion has no overloaded operator, but is created using a `PointCutExpressionNot` node. An example of a point cut expression and its corresponding AST can be seen in Figure S3.

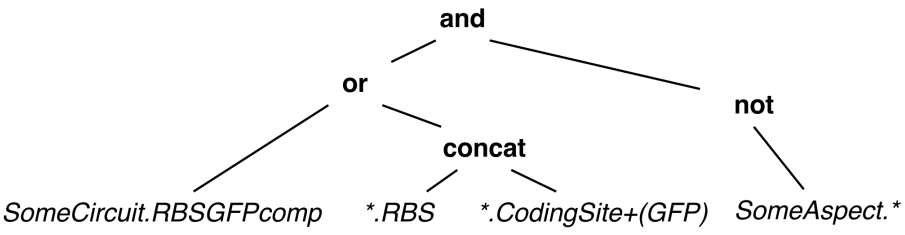

Figure S3: An AST of a point cut expression: `( (PartSelector(‘SomeCircuit.RBSGFPcomp’) | (PartSelector(‘*.RBS’) % PartSelector(‘*.CodingSite+.(GFP)) & PointCutExpressionNot(PartSelector(‘SomeAspect.*’))`

1.5 Aspects and Advice

Advice is the code that should be inserted at join points matched by point cuts. For SynBioWeaver, this means that advice should also be able to use `addPart()`. An advice is bundled into an *aspect*. Each aspect is a subclass of the abstract `Aspect` class, and must implement a `mainAspect()` method, in which all advice is declared, using `addAdvice()`. This takes three parameters: a point cut, a method of the aspect that is to be called at the appropriate join point, and an optional precedence parameter. Advice methods belong to the corresponding aspect, and thus can access aspect attributes via `self`. They also receive a `PointCutContext` parameter that can access the matched part and “advice stack” information.

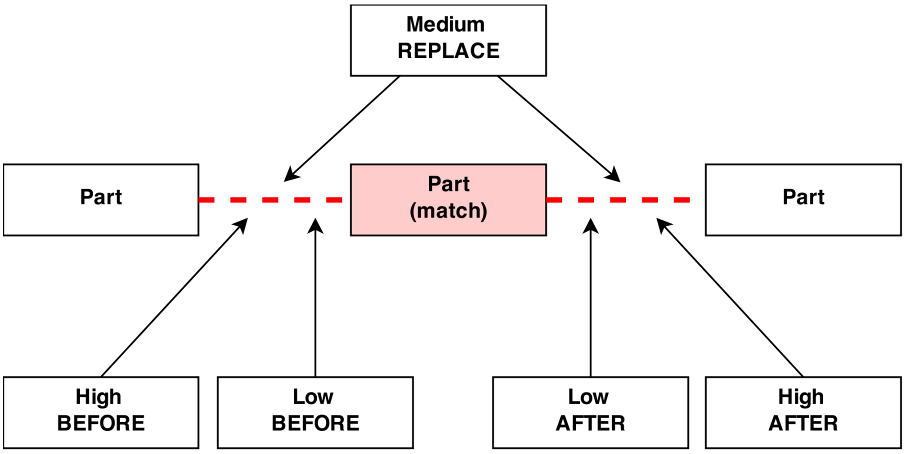

Figure S4: Five point cut / advice pairs of different operators and precedence match the same part. The low precedence *before* and *after* advice will not be executed, because of the medium precedence *replace* advice.

1.5.1 Advice precedence

If more than one point cut matches a join point, order of advice execution is undefined unless precedence is explicitly set via the third parameter to `addAdvice()`. `precedence` is an integer between `Advice.MINPRECEDENCE` and `Advice.MAXPRECEDENCE`. By default, every advice is set to minimal precedence. If two advices have the same precedence, execution order is also undefined. For two advices sharing a *before* point cut, the higher precedence advice will execute **before** the lower precedence advice. Thus, if both advices add a part, the part from the high precedence advice will be first in the resulting circuit. Similarly, for two advices sharing an *after* point cut, the higher precedence advice will execute **after** the low precedence advice. These precedence rules are similar to precedence in AspectJ. For two advices sharing a *replace* point cut, the higher precedence advice will execute, whereas all lower advices will be ignored. This includes lower precedence *before* and *after* advices. However, *after* advices with similar or higher precedence will still be executed. Figure S4 exemplifies the precedence rules.

1.6 Type Advice

Apart from normal advice, which changes the execution flow, it is also possible to modify the `Molecule` and `Part` types using type advice. For example, one aspect could add sequence information to a whole range of parts; another could add modelling rules to parts based on features selected by a `PartSignature`. As a demonstration, below is a simple example to add a method to Promoter types to print if they are regulated or not.

Listing 3: A simple example of using type advice

```
from synbioweaver import *


class TwoPromoters(Circuit):
    def mainCircuit(self):
        self.addPart(Promoter)
        self.createMolecule(Protein)
        self.addPart(NegativePromoter(Protein))

class PromoterTypeAdvice(Aspect):
    def mainAspect(self):
        # define three signatures
        nonRegulatedPromoterSignature = PartSignature('*.Promoter+()')
        regulatedPromoterSignature = PartSignature('*.Promoter+(Molecule+)')
```

```
        anyPromoterSignature = PartSignature('*.Promoter+')

        # add Boolean Attribute "isRegulated" to two different types of Promoters
        self.addTypeAdvice(nonRegulatedPromoterSignature,False,"isRegulated")
        self.addTypeAdvice(regulatedPromoterSignature,True,"isRegulated")
        self.addTypeAdvice(MoleculeSignature('*.*'),4,"valueNumber")
        self.addTypeAdvice(MoleculeSignature('*.*'),self.printNumber,"printValue")

        # add a type advice method
        self.addTypeAdvice(anyPromoterSignature,self.printIsRegulated,"printIsRegulated")

    def printNumber(self,mol):
        print "molecule value is "+str(mol.valueNumber)

    def printIsRegulated(self,part):
        if part.isRegulated == False:
            print str(part)+" is not regulated by a Molecule."
        else:
            print str(part)+" is regulated by a Molecule."

compiledDesign = Weaver(TwoPromoters,PromoterTypeAdvice).output()
for part in compiledDesign.partList:
    part.printIsRegulated()

for molecule in compiledDesign.moleculeList:
    molecule.printValue()
```

This give the output:

```
Promoter is not regulated by a Molecule.
NegativePromoter(regulatedBy = Protein) is regulated by a Molecule.
molecule value is 4
```

1.7 Weaving

The `Weaver` object represents the SynBioWeaver "compiler", taking a single `Circuit` class (which can use other circuits as composites) and a number of `Aspect` classes. Advice from aspects is automatically woven into the part based execution flow, at join points matched by point cuts, resulting in a single sorted list of parts. Figure S5 shows a sequence diagram demonstrating weaving a single before advice into a circuit.

Schematically, this is how the Weaver works:

- Call each aspect's `mainAdvice()` method and construct a list of advice, including type advice and advice defining additional output targets for the weaver.
- Sort before, replace and after advice lists according to precedence.
- Begin circuit construction by calling the `Circuit` `mainCircuit()` method.
- At some point, `mainCircuit()` will call the Weaver's `addPart()` method. At this point, advice might be inserted, which itself may call `addPart()`. Thus, this process is recursive. `addPart()` proceeds as follows:
  - Initialise the part and add any type advice.
  - If any before or replace advice have matching point cuts, run these.
  - Unless replaced, append the part to the list of parts.
  - Run any remaining after advice that have matching point cuts and appropriate precedence.
- After the outermost `addPart()` call and `mainCircuit()` has returned, create a `WeaverOutput` object to hold the results, including the list of parts and a list of involved molecules.

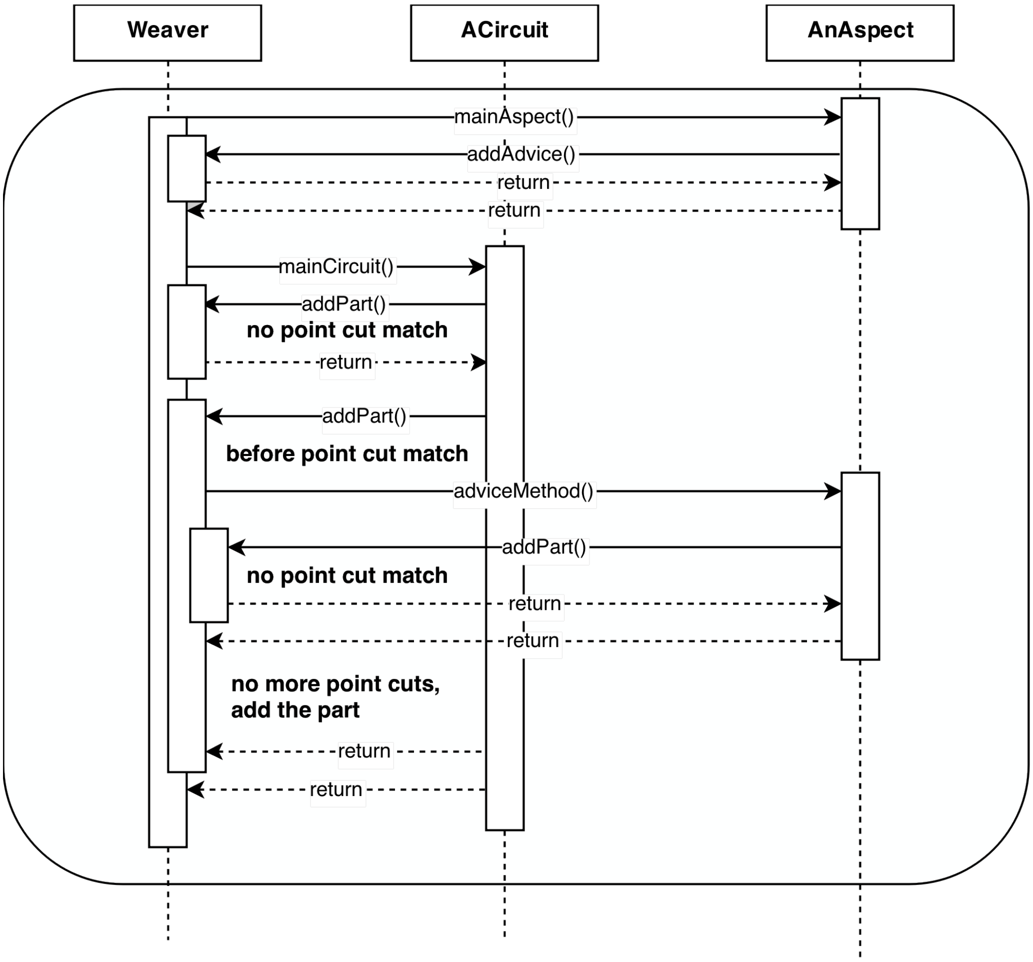

Figure S5: The Weaver "compiles" an aspect and a circuit. In this example, a before point cut from the aspect is satisfied and triggered, leading to a single part being inserted into the circuit.

2 A switchable oscillating system based on the repressilator combined with an AND gate

In this example, we demonstrate how to build an oscillating GFP output from component part designs. We consider the following concerns:

- Oscillation (using abstract, non-determined transcription factors)
- Terminator and RBS Design Rules (as seen in 1.2 on page 2)
- Two output versions: Just via coding GFP, or alternatively, coding GFP dependent an external inducer (that is, an additional on-switch)

A concern for the instantiation of abstract molecules is also included in the example in the package.

2.1 Defining the concerns

First, here is a possible implementation of the two GFP coding concerns:

Listing 4: Repressilator Example: GFP Coding

```
# GFP circuit definitions
```

```
declareNewMolecule('exIn')
declareNewMolecule('GFP')
declareNewPart('cGFP', CodingRegion, moleculesAfter=[GFP] )

class CodingGFP(Circuit):
    # constitutively code gfp
    def mainCircuit(self):
        declareNewPart('Pconst',ConstitutivePromoter)
        self.addPart(Pconst)
        self.addPart(cGFP)

class InduceGFP(Circuit):
    # code GFP dependent on an external inducer

    def getSignal(self):
        return exIn

    def getOutputName():
        return cGFP

    def mainCircuit(self):
        self.createMolecule(exIn)
        declareNewPart('Pin', PositivePromoter, [exIn] )
        self.addPart(Pin)
        self.addPart(cGFP)
```

Next, we define a composite circuit that can create oscillating behaviour. It consists of three promoter and coding region pairs, where each coded protein represses the promoter for the next coding region[2].

Listing 5: Repressilator Example: Abstract Repressilator Circuit

```
class AbstractRepressilatorComposite(Circuit):
    def getSignal(self):
        return A

    def mainCircuit(self):
        declareNewMolecule('A')
        declareNewMolecule('B')
        declareNewMolecule('C')
        self.createMolecule(A)
        self.createMolecule(B)
        self.createMolecule(C)

        declareNewPart('p1', NegativePromoter, [C] )
        declareNewPart('p2', NegativePromoter, [A] )
        declareNewPart('p3', NegativePromoter, [B] )
        declareNewPart('cA', CodingRegion, moleculesAfter=[A] )
        declareNewPart('cB', CodingRegion, moleculesAfter=[B] )
        declareNewPart('cC', CodingRegion, moleculesAfter=[C] )
        self.addPart( p1 )
        self.addPart( cA )
        self.addPart( p2 )
        self.addPart( cB )
        self.addPart( p3 )
```

[2]The design is based on Elowitz and Leibler (2000), with transcription factors made abstract. They will later be re-instantiated using another aspect.

```
        self.addPart( cC )
```

Now, we design an aspect that can weave oscillation into either of the two coding circuits. This means, the aspect needs to be able to change the promoter of the coding circuit to make coding dependent on oscillation. If the promoter is not regulated, it must be replaced by one that is regulated by oscillation output. However, if the promoter is already regulated, we need to insert an additional And-Gate to preserve the original input[3].

Listing 6: Repressilator Example: Repressilation aspect

```
class Repressilation(Aspect):
    # weave repressilator into a one-protein coding circuit
    def mainAspect(self):
        self.repressilatorComposite = AbstractRepressilatorComposite()

        # promoter is unregulated (not from this aspect or the composite)
        unregulatedPromoterReplace = PointCut(
            PartSignature('!AbstractRepressilatorComposite.Promoter+()') &
            PartSignature('!Repressilation.Promoter+()'),PointCut.REPLACE)
        self.addAdvice(unregulatedPromoterReplace, self.insertRegulatedBySignalPromoter, 10)

        # regulated case
        regulatedCodingExpressionReplace = PointCut(
            (PartSignature('InduceGFP.PositivePromoter+') &
             PartSignature('!AbstractRepressilatorComposite.Promoter+(Molecule+)') &
             PartSignature('!Aspect+.Promoter+(Molecule+)') &
             PartSignature('!AndGate.Promoter+(Molecule+)')) % PartSignature('*.CodingRegion+(Molecule+)'),
            PointCut.REPLACE)
        self.addAdvice(regulatedCodingExpressionReplace, self.insertBooleanAndSignal, 10)

        # add repressilatior composite
        promoterAndCodingRegion = PartSignature('Repressilation.Promoter+') % PartSignature('*.RBS+') % PartSignature('*.CodingRegion+') % PartSignature('*.Terminator+')
        andGateRegion = PartSignature('Repressilation.AndGate')
        afterCircuit = PointCut(andGateRegion | promoterAndCodingRegion, PointCut.AFTER)
        self.addAdvice(afterCircuit, self.insertComposite, 10)

    def insertRegulatedBySignalPromoter(self,context):
        signal = self.repressilatorComposite.getSignal()
        context.within[0].createMolecule( signal )
        declareNewPart('ppA', PositivePromoter, [signal] )
        self.addPart(ppA)
        return True

    def insertBooleanAndSignal(self,context):
        originalpromoter = context.part.before[0]
        originalsignal = context.part.before[0].getRegulatedBy()[0]
        originaloutput = context.part.getCodingFor()[0]
        andGate = AndGate()

        # Creat first input for AND gate
        context.within[0].createMolecule( exIn )
```

[3]The code of the And-Gate Circuit is not reproduced here but can be found in the complete code of the example. The design is based on Wang et al. (2011).

```
        andGate.moleculeInputOne = exIn

        if isinstance(originalpromoter,PositivePromoter):
            andGate.setInputOneRepressing = False
        else:
            andGate.setInputOneRepressing = True

        # Create second input for AND gate
        in2 = self.repressilatorComposite.getSignal()
        context.within[0].createMolecule( in2 )
        andGate.moleculeInputTwo = in2

        andGate.setInputOneRepressing = True
        andGate.moleculeOutput = originaloutput

        self.addPart(andGate)
        return True

    def insertComposite(self,context):
        context.within[0].createMolecule( A )
        context.within[0].createMolecule( B )
        context.within[0].createMolecule( C )
        self.addPart(self.repressilatorComposite)
        return True
```

3 Rule-based modelling as an output concern

3.1 Overview

To demonstrate how a rule-based model could be generated as an output concern from a genetic circuit, we implemented an aspect that annotates the genetic parts in the system with appropriate reaction rules, which can then be used in a stochastic simulation or to generate ODEs. The reaction rules were based on a Kappa model of the Repressilator by Stewart and Wilson-Kanamori (2011) and include:

- Transcription Factor Binding and Un-Binding to Promoter parts
- RNAP binding to Promoter parts based on various configurations of transcription factors.
- RNAP Progression (Transcription) from Promoter parts downstream until Terminator parts resulting in an mRNA part chain
- Ribosome binding to mRNA RBS part
- Ribosome translation of the Protein Coding Region
- Protein Degradation
- mRNA Degradation

3.2 Defining the Concerns

The Repressilator was encoded in the circuit with parts named after their appropriate counterparts in the Parts Registry.

Listing 7: Extract from the PYSBmodel aspect

```
# transcription factor used in the repressilator system
declareNewMolecule('LacI')
declareNewMolecule('TetR')
declareNewMolecule('lCl')

declareNewPart('BBaB0011',Terminator)
declareNewPart('BBaB0034',RBS)
declareNewPart('BBaC0012',CodingRegion,moleculesAfter=[LacI])
declareNewPart('BBaC0040',CodingRegion,moleculesAfter=[TetR])
declareNewPart('BBaC0051',CodingRegion,moleculesAfter=[lCl])
declareNewPart('BBaR0010',NegativePromoter,moleculesBefore=[LacI])
declareNewPart('BBaR0040',NegativePromoter,moleculesBefore=[TetR])
declareNewPart('BBaR0051',NegativePromoter,moleculesBefore=[lCl])


declareNewMolecule('GFP')

class Repressilator(Circuit):
    def mainCircuit(self):
        self.createMolecule(LacI)
        self.addPart(BBaR0010)
        self.addPart(BBaC0040)
        self.addPart(BBaR0040)
        self.addPart(BBaC0051)
        self.addPart(BBaR0051)
        self.addPart(BBaC0012)
```

The rule-based modelling concern is implemented using a parameterized aspect, so that all of the parameters used in the model can be easily customized. The default values used in the example follow the model by Stewart and Wilson-Kanamori (2011).

Listing 8: Repressilator Circuit Specification

```
class PYSBmodel(Aspect):
    observableProteins = []

    def __init__(self,rnapCount = 700,ribosomeCount = 18000,operonCount = 1,
                 rnapBindingHigh = 0.0007,rnapBindingLow=7e−07,
                 transcriptionRate = 10,transcriptionFactorBinding = 0.01,
                 transcriptionFactorUnbinding = 0.09,transcriptionFactorUnbindingSole
                     =2.24,
                 transcriptionFactorFactorDegradation=0.00115,rbsBinding = 0.000166,
                 translationInitiationRate=0.167,translationRate=10,ribosomeFalloff =
                     0.01,
                 rnapFalloffRate=1,rnaDegradation=0.0058):
```

The rules and model are implemented in PySB, a python based rule-based modelling language (Lopez et al., 2013). When the aspect is initialized, it creates a PySB model and annotates each part in the system with specific type advice. For example, a PySB monomer for a part's DNA piece and potential RNA piece are added to the model. Additionally, the necessary reaction rules are added to the model, according to the parts in the system. A Terminator's rule is relatively straightforward:

Listing 9: PYSB rule for Transcription Termination

```
            pysb.Rule('Transcription_Termination_'+ part.pysbname,
                          self.DNA(binding = 1, partType = part.pysbname) +
```

```
                            self.RNAP(dna = 1,rna = 2) +
                            self.RNA(downstream = 2)
                            >>
                            self.DNA(binding = None, partType = part.pysbname) +
                            self.RNAP(dna = None,rna = None) +
                            self.RNA(downstream = None),
                            self.TranscriptionRate)
```

On the other hand, the whole method to add rules for a Negative Promoter with two binding sites is quite long:

Listing 10: PYSB rule for Transcription Termination

```
    def pysbPartRulesNegPromoter(self,part):
        regulatorMol = part.getBeforeNodes(Molecule)[0]

        # transcription factor binding
        pysb.Rule('TF_Binding_to_' + part.pysbname + 'p2' + '_noTF',
                self.DNA(binding = None,partType = part.pysbname + 'p3',upstream = 2) +
                regulatorMol.pysbmonomer(dna = None) +
                self.DNA(downstream = 2,binding = None,partType = part.pysbname + 'p2')
                <>
                self.DNA(binding = None,partType = part.pysbname + 'p3',upstream = 3) +
                regulatorMol.pysbmonomer(dna = 1) +
                self.DNA(downstream = 3,binding = 1,partType = part.pysbname + 'p2'),
                self.TranscriptionFactorBinding, self.TranscriptionFactorUnbindingSole)

        pysb.Rule('TF_Binding_to_' + part.pysbname + 'p2' + '_TF',
                self.DNA(binding = 1,partType = part.pysbname + 'p3',upstream = 2) +
                regulatorMol.pysbmonomer(dna = 1) +
                self.DNA(downstream = 2,binding = None,partType = part.pysbname + 'p2') +
                regulatorMol.pysbmonomer(dna = None)
                <>
                self.DNA(binding = 2,partType = part.pysbname + 'p3',upstream = 3) +
                regulatorMol.pysbmonomer(dna = 2) +
                self.DNA(downstream = 3,binding = 1,partType = part.pysbname + 'p2') +
                regulatorMol.pysbmonomer(dna = 1),
                self.TranscriptionFactorBinding,self.TranscriptionFactorUnbinding)

        pysb.Rule('TF_Binding_to_' + part.pysbname + 'p3' + '_noTF',
                self.DNA(binding = None,partType = part.pysbname + 'p3',upstream = 2) +
                regulatorMol.pysbmonomer(dna = None) +
                self.DNA(downstream = 2,binding = None,partType = part.pysbname + 'p2')
                <>
               self.DNA(binding = 1,partType = part.pysbname + 'p3',upstream = 3) +
                regulatorMol.pysbmonomer(dna = 1) +
                self.DNA(downstream = 3,binding = None,partType = part.pysbname + 'p2'),
                self.TranscriptionFactorBinding,self.TranscriptionFactorUnbindingSole)

        pysb.Rule('TF_Binding_to_' + part.pysbname + 'p3' + '_TF',
                self.DNA(binding = None,partType = part.pysbname + 'p3',upstream = 2) +
                regulatorMol.pysbmonomer(dna = 1) +
                self.DNA(downstream = 2,binding = 1,partType = part.pysbname + 'p2') +
                regulatorMol.pysbmonomer(dna = None)
                <>
                self.DNA(binding = 1,partType = part.pysbname + 'p3',upstream = 3) +
                regulatorMol.pysbmonomer(dna = 2) +
```

```
                self.DNA(downstream =3,binding = 2,partType = part.pysbname + 'p2') +
                regulatorMol.pysbmonomer(dna = 1),
                self.TranscriptionFactorBinding,self.TranscriptionFactorUnbinding)

        # RNAP binding
        pysb.Rule('RNAP_Binding_To_' + part.pysbname+'_no_TF',
                self.DNA(binding = None,partType = part.pysbname + 'p3',upstream = 2,
                    downstream = 1) +
                self.DNA(binding = None,partType = part.pysbname + 'p4',upstream = 1) +
                self.RNAP(dna = None,rna = None) +
                self.DNA(binding = None,partType = part.pysbname + 'p2',downstream = 2)
                >>
               self.DNA(binding = None,partType = part.pysbname + 'p3',upstream = 3,
                   downstream = 1) +
                self.DNA(binding = 2,partType = part.pysbname + 'p4',upstream = 1) +
                self.RNAP(dna = 2,rna = None) +
                self.DNA(binding = None,partType = part.pysbname + 'p2',downstream = 3),
                self.RNAPBindingHigh)

        pysb.Rule('RNAP_Binding_To_' + part.pysbname+'_TF_on_p2',
                self.DNA(binding = None,partType = part.pysbname + 'p3',upstream = 2,
                    downstream = 1) +
                self.DNA(binding = None,partType = part.pysbname + 'p4',upstream = 1) +
                self.RNAP(dna = None,rna = None) +
                self.DNA(binding = 3,partType = part.pysbname + 'p2',downstream = 2) +
                regulatorMol.pysbmonomer(dna = 3)
                >>
                self.DNA(binding = None,partType = part.pysbname + 'p3',upstream = 3,
                    downstream = 1) +
                self.DNA(binding = 2,partType = part.pysbname + 'p4',upstream = 1) +
                self.RNAP(dna = 2,rna = None) +
                self.DNA(binding = 4,partType = part.pysbname + 'p2',downstream = 3) +
                regulatorMol.pysbmonomer(dna = 4),
                self.RNAPBindingLow)

        pysb.Rule('RNAP_Binding_To_' + part.pysbname+'_TF_on_p3',
                self.DNA(binding = 3,partType = part.pysbname + 'p3',upstream = 2,
                    downstream = 1) +
                self.DNA(binding = None,partType = part.pysbname + 'p4',upstream = 1) +
                self.RNAP(dna = None,rna = None) +
                self.DNA(binding = None,partType = part.pysbname + 'p2',downstream = 2) +
                regulatorMol.pysbmonomer(dna = 3)
                >>
                self.DNA(binding = 4,partType = part.pysbname + 'p3',upstream = 3,
                    downstream = 1) +
                self.DNA(binding = 2,partType = part.pysbname + 'p4',upstream = 1) +
                self.RNAP(dna = 2,rna = None) +
                self.DNA(binding = None,partType = part.pysbname + 'p2',downstream = 3) +
                regulatorMol.pysbmonomer(dna = 4),
                self.RNAPBindingLow)

        pysb.Rule('RNAP_Binding_To_' + part.pysbname+'_TF_on_p2_and_p3',
                self.DNA(binding = 3,partType = part.pysbname + 'p3',upstream = 2,
                    downstream = 1) +
                self.DNA(binding = None,partType = part.pysbname + 'p4',upstream = 1) +
```

```
                self.RNAP(dna = None,rna = None) +
                self.DNA(binding = 4,partType = part.pysbname + 'p2',downstream = 2) +
                regulatorMol.pysbmonomer(dna = 3) +
                regulatorMol.pysbmonomer(dna = 4)
                >>
                self.DNA(binding = 4,partType = part.pysbname + 'p3',upstream = 3,
                    downstream = 1) +
                self.DNA(binding = 2,partType = part.pysbname + 'p4',upstream = 1) +
                self.RNAP(dna = 2,rna = None) +
                self.DNA(binding = 5,partType = part.pysbname + 'p2',downstream = 3) +
                regulatorMol.pysbmonomer(dna = 4) +
                regulatorMol.pysbmonomer(dna = 5),
                self.RNAPBindingLow)

        # Transcription
        pysb.Rule('Transcription_Initiation_Of_'+part.pysbname,
                self.DNA(binding = 1,partType = part.pysbname + 'p4',downstream = 2) +
                self.RNAP(dna = 1,rna = None) +
                self.DNA(upstream = 2,binding = None)
                >>
                self.DNA(binding = None,partType = part.pysbname + 'p4',downstream = 3) +
                self.RNAP(dna = 1,rna = 2) +
                self.DNA(upstream = 3,binding = 1) +
                self.RNA(binding = None,upstream = None,downstream = 2,partType = part.
                    pysbname),
                self.TranscriptionRate)
```

PySB was chosen because of its availability for Python and because it can create ODEs from the model or act as a translator to other established rule-based modelling languages such as Kappa or BNG.

4 Generating reaction network models and adding context

To demonstrate weaving at the model level, we have implemented a number of aspects for reaction network generation and contextual cross-cutting concerns. We chose to separate model generation from contextual concerns to enable consistency when called from the `WeaverOutput` object.

The current model generation aspects are as follows:

- `PromoterMapping` - this is required to map coding regions to promoters
- `MassActionKineticsProtein` - assumes mass action kinetics including only protein level abstraction
- `MassActionKineticsRNA` - assumes mass action kinetics including both protein and mRNA level abstraction
- `SheaAckersKineticsRNA` - assumes Shea-Ackers equilibrium dynamics at the promoters and mass action kinetics otherwise, including both protein and mRNA level abstraction

These aspects add the method `getReactions` to the `WeaverOutput` object. All downstream aspects that require the reactions generated must check that this method exists using `getattr(weaverOutput, "getReactions")`. On the other hand, design contextual aspects call `getReactions` but add the method `getContext`. Examples of these are

- `LagLogisticGrowth`
- `PostTranslationalCoupling`

which are described in the next two sections. Downstream analysis aspects should always use the `getContext` method over the `getReactions` method to ensure that the most up-to-date model is used. Examples of these are

- `PrintReactionNetwork`
- `WriteSBMLModel`

This is one possible choice of constraining how aspects interact at the model level and there may be other ways. One restriction of this approach is that only one model generation aspect, and one contextual aspect can be called. In future developments of the framework it would be advantageous to allow multiple reaction and contextual aspects.

5 Bayesian modelling and characterisation

5.1 Code for constitutive GFP and different bacterial growth contexts

In this example a simple model for the constitutive expression of GFP (core concern) is combined with the environmental context (cross-cutting concern) of bacterial growth. This example also shows how SBML models can be generated under different contexts.

Listing 11: Adding a bacterial growth cross-cutting concern

```
from synbioweaver.aspects.writeCudaFileODE import *
from synbioweaver.aspects.writeABCInputFileODE import *
from synbioweaver.aspects.writeCudaFileGillespie import *
from synbioweaver.aspects.writeABCInputFileGillespie import *

declareNewMolecule('GFP')

class constGFP(Circuit):
    def mainCircuit(self):
        # constitutive promoter
        self.addPart(ConstitutivePromoter)
        self.addPart(CodingRegion(GFP))

print "####### circuit + exp growth"
compiledDesign1 = Weaver(constGFP, DesignRules, PromoterMapping, MassActionKineticsProtein, ExpGrowth, PrintReactionNetwork, WriteSBMLModel).output()
compiledDesign1.printReactionNetwork()
modelStr = compiledDesign1.writeSBMLModel()

# write an SBML model out
sbmlFile = open("constGFP_exp.sbml","w")
print >>sbmlFile, modelStr
sbmlFile.close()

print "####### circuit + logistic growth"
compiledDesign2 = Weaver(constGFP, DesignRules, PromoterMapping, MassActionKineticsProtein, LogisticGrowth, PrintReactionNetwork, WriteSBMLModel).output()
compiledDesign2.printReactionNetwork()
modelStr = compiledDesign2.writeSBMLModel()

# write an SBML model out
sbmlFile = open("constGFP_log.sbml","w")
```

```
print >>sbmlFile, modelStr
sbmlFile.close()

print "####### circuit + lag logistic growth"
compiledDesign3 = Weaver(constGFP, DesignRules, PromoterMapping,
    MassActionKineticsProtein, LagLogisticGrowth, PrintReactionNetwork, WriteSBMLModel).
    output()
compiledDesign3.printReactionNetwork()
modelStr = compiledDesign3.writeSBMLModel()

# write an SBML model out
sbmlFile = open("constGFP_lag.sbml","w")
print >>sbmlFile, modelStr
sbmlFile.close()
```

5.2 Model for the core concern plus lag logistic growth

The model for the core concern is a basic birth death system generated using the `MassActionKineticsProtein` aspect.

$$\begin{aligned} \emptyset &\rightarrow GFP & \quad & p_1 \\ GFP &\rightarrow \emptyset & & p_2 GFP \end{aligned}$$

The contextual aspect `LagLogisticGrowth` models bacterial growth using a two population system (Yates and Smotzer, 2007). N represents bacteria that divide and N' represent bacteria that do not divide and eventually die. A transition between the two states is included to model bacteria that are initially non dividing but after a delay they start dividing. After the cross-cutting concern is included the model becomes

$$\begin{aligned} N &\rightarrow N + GFP & \quad & p_1 N \\ GFP &\rightarrow \emptyset & & p_2 GFP \\ N &\rightarrow 2N & & p_3(1 - N/p_4)N \\ N' &\rightarrow \emptyset & & p_5 N' \\ N' &\rightarrow N & & p_6 N' \end{aligned}$$

6 Post-translational coupling of a bistable switch and an oscillator

6.1 Code for the combined and coupled systems

The code for the combined system where proteins AraC and A are tagged is shown below.

Listing 12: Post-translational coupling of a bistable with and an oscillator. Coupling proteins AraC and A

```
declareNewMolecule('GFP')
declareNewMolecule('LacI')
declareNewMolecule('AraC_tag')
declareNewMolecule('LacIn4')
declareNewMolecule('AraCn2')
declareNewMolecule('zero')

declareNewPart("P1", HybridPromoter, [LacIn4, AraCn2], regulatorInfoMap={LacIn4:False,
    AraCn2:True})
```

```
declareNewPart("P2", HybridPromoter, [LacIn4, AraCn2], regulatorInfoMap={LacIn4:False,
    AraCn2:True})
declareNewPart("P3", HybridPromoter, [LacIn4, AraCn2], regulatorInfoMap={LacIn4:False,
    AraCn2:True})

declareNewMolecule('A_tag')
declareNewMolecule('B')
declareNewMolecule('An2')
declareNewMolecule('Bn2')
declareNewMolecule('In')

declareNewPart('Pa', NegativePromoter, [An2])
declareNewPart('Pb', NegativePromoter, [Bn2])

class S(Circuit):
    def mainCircuit(self):

        self.createMolecule(GFP)
        self.createMolecule(LacI)
        self.createMolecule(AraC_tag)
        self.createMolecule(LacIn4)
        self.createMolecule(AraCn2)

        self.addPart(P1)
        self.addPart(CodingRegion(AraC_tag))

        self.addPart(P2)
        self.addPart(CodingRegion(LacI))

        self.addPart(P3)
        self.addPart(CodingRegion(GFP))

        self.reactionFrom(LacI, LacI, LacI, LacI) >> self.reactionTo( LacIn4 )
        self.reactionFrom(LacIn4) >> self.reactionTo( zero )
        self.reactionFrom(AraC_tag, AraC_tag) >> self.reactionTo( AraCn2 )
        self.reactionFrom(AraCn2) >> self.reactionTo( zero )

class T(Circuit):
    def mainCircuit(self):

        self.createMolecule(A_tag)
        self.createMolecule(An2)
        self.createMolecule(B)
        self.createMolecule(Bn2)
        self.createMolecule(In)

        self.addPart(Pa)
        self.addPart(CodingRegion(B))
        self.addPart(Pb)
        self.addPart(CodingRegion(A_tag))

        self.reactionFrom(A_tag, A_tag) >> self.reactionTo( An2 )
        self.reactionFrom(B, B) >> self.reactionTo( Bn2 )
        self.reactionFrom(An2) >> self.reactionTo( zero )
        self.reactionFrom(Bn2) >> self.reactionTo( zero )
```

```
        self.reactionFrom(zero) >> self.reactionTo( In )
        self.reactionFrom(In,Bn2) >> self.reactionTo( In )

class toggleOsc(Circuit):
    def mainCircuit(self):
        self.addCircuit(S)
        self.addCircuit(T)


compiledDesign = Weaver(toggleOsc, DesignRules, PromoterMapping, SheaAckersKineticsRNA,
    PostTranslationalCoupling, PrintReactionNetwork).output()
compiledDesign.printReactionNetwork()
```

The models, given below, are generated using the SheaAckersKineticsRNA aspect. The aspect PostTranslationalCoupling identifies proteins with names ending in _tag and assigns them an enzymatic degradation term.

6.2 Toggle switch model

The numbers of the the two proteins, A and B, are represented by A and B respectively and their mRNA transcripts by mA and mB. We assume that both proteins form dimers. The number of inducer molecules is represented by In.

$$
\begin{aligned}
\emptyset &\to mB & p_3p_1/(1+p_1+p_2A_2) \\
B &\to \emptyset & p_7B \\
mB &\to \emptyset & p_8mB \\
mB &\to B & p_9mB \\
\emptyset &\to mA & p_6p_4/(1+p_4+p_5B_2) \\
A &\to \emptyset & p_{10}A \\
mA &\to \emptyset & p_{11}mA \\
mA &\to A & p_{12}mA \\
2A &\to A_2 & p_{13}A^2 \\
A_2 &\to \emptyset & p_{14}A_2 \\
2B &\to B_2 & p_{15}B^2 \\
B_2 &\to \emptyset & p_{16}B_2 \\
\emptyset &\to In & p_{17} \\
In + B_2 &\to In & p_{18}B_2
\end{aligned}
$$

6.3 Stricker oscillator model

The numbers of the AraC, LacI and GFP proteins are represented by X, Y and G respectively and their mRNA transcripts by mX, mY and mG.

$$
\begin{aligned}
\emptyset \rightarrow mX & \qquad p_4(p_1 + p_3 X_2)/(1 + p_1 + p_2 Y_4 + p_3 X_2) \\
X \rightarrow \emptyset & \qquad p_{13} X \\
mX \rightarrow \emptyset & \qquad p_{14} mX \\
mX \rightarrow X & \qquad p_{15} mX \\
\emptyset \rightarrow mY & \qquad p_8(p_5 + p_7 X_2)/(1 + p_5 + p_6 Y_4 + p_7 X_2) \\
Y \rightarrow \emptyset & \qquad p_{16} Y \\
mY \rightarrow \emptyset & \qquad p_{17} mY \\
mY \rightarrow X & \qquad p_{18} mY \\
\emptyset \rightarrow mG & \qquad p_{12}(p_9 + p_{11} X_2)/(1 + p_9 + p_{10} Y_4 + p_{11} X_2) \\
G \rightarrow \emptyset & \qquad p_{19} G \\
mG \rightarrow \emptyset & \qquad p_{20} mG \\
mG \rightarrow G & \qquad p_{21} mG \\
4Y \rightarrow Y_4 & \qquad p_{22} Y^4 \\
Y_4 \rightarrow \emptyset & \qquad p_{23} Y_4 \\
2X \rightarrow X_2 & \qquad p_{24} X^2 \\
X_2 \rightarrow \emptyset & \qquad p_{25} X_2
\end{aligned}
$$

References

M. B. Elowitz and S. Leibler. A synthetic oscillatory network of transcriptional regulators. *Nature*, 403(6767): 335–338, Jan. 2000. doi: 10.1038/35002125.

C. F. Lopez, J. L. Muhlich, J. A. Bachman, and P. K. Sorger. Programming biological models in Python using PySB. *Molecular Systems Biology*, 9:646, 2013. doi: 10.1038/msb.2013.1.

D. Stewart and J. R. Wilson-Kanamori. Modular modelling in synthetic biology: Light-based communication in e. coli. *Electr. Notes Theor. Comput. Sci.*, 277:77–87, 2011.

B. Wang, R. I. Kitney, N. Joly, and M. Buck. Engineering modular and orthogonal genetic logic gates for robust digital-like synthetic biology. *Nature Communications*, 2:508, 2011.

G. T. Yates and T. Smotzer. On the lag phase and initial decline of microbial growth curves. *Journal of theoretical biology*, 244(3):511–517, Feb. 2007. doi: 10.1016/j.jtbi.2006.08.017.
